# Synergistic enhancement of tendon-to-bone healing via anti-inflammatory and pro-differentiation effects caused by sustained release of Mg^2+^/curcumin from injectable self-healing hydrogels

**DOI:** 10.7150/thno.56266

**Published:** 2021-04-03

**Authors:** Baojun Chen, Yongping Liang, Jing Zhang, Lang Bai, Meiguang Xu, Qian Han, Xuezhe Han, Jintao Xiu, Meng Li, Xiaoling Zhou, Baolin Guo, Zhanhai Yin

**Affiliations:** 1 Department of Orthopaedics, The First Affiliated Hospital of Xi'an Jiaotong University, Xi'an, 710061, China.; 2 Frontier Institute of Science and Technology, and State Key Laboratory for Mechanical Behavior of Materials, and Key Laboratory of Shaanxi Province for Craniofacial Precision Medicine Research, College of Stomatology, Xi'an Jiaotong University, Xi'an, 710049, China.; 3 Key Laboratory of Resource Biology and Biotechnology in Western China, Ministry of Education. School of Medicine, Northwest University, 229 Taibai North Road, Xi'an 710069, China.

**Keywords:** anti-inflammation, pro-chondrogenesis, hydrogels, tendon-to-bone healing, rotator cuff, synergistic effects

## Abstract

Poor healing response after rotator cuff reconstruction is multifactorial, with the inflammatory microenvironment and deficiency of stem cell differentiation factors at the lesion site being most relevant. However, there is a lack of effective tissue engineering strategies that can simultaneously exert anti-inflammatory and pro-differentiation effects to promote rotator cuff healing.

**Methods:** In this study, we synthesized and characterized a novel active drug delivery vector that successfully overcame the challenge of simultaneous high-efficiency loading and controlled release of Mg^2+^ and curcumin. The anti-inflammatory and pro-differentiation effects of the composite hydrogel were evaluated* in vitro* and *in vivo*. Moreover, healing of the rotator cuff tendon-to-bone interface was studied by histology, immunofluorescence, and biomechanical tests.

**Results:** The composite hydrogel exhibited excellent biocompatibility and injectability, good adhesiveness, and rapid self-healing. The released curcumin showed obvious anti-inflammatory and antioxidation effects, which protected stem cells and tendon matrix. Furthermore, released Mg^2+^ promoted stem cell aggregation and chondrogenesis. Moreover, biomechanical tests and histological results of a rat rotator cuff tear model at 8 weeks after surgery indicated that the composite hydrogel significantly enhanced tendon-to-bone healing.

**Conclusions:** The composite hydrogel mediated sustained *in situ* release of curcumin and Mg^2+^ to effectively promote rotator cuff tendon-to-bone healing via anti-inflammatory and pro-differentiation effects. Therefore, this composite hydrogel offers significant promise for rotator cuff repair.

## Introduction

Rotator cuff tear is a common shoulder disease with an incidence of more than 20% that frequently requires surgical reconstruction 1, 2. The current surgical approaches to repair torn rotator cuff tendons have unacceptably low success ratios 3. Failure is ascribed to a lack of functional enthesis structure, consisting of tendon, non-calcified fibrocartilage, calcified fibrocartilage, and bone, and formation of fibrovascular scar tissue with inferior mechanical properties 4. Various tissue engineering strategies have been developed to promote tendon-to-bone healing and have demonstrated potential for functional re-integration of the injured rotator cuff 5-8. However, it remains challenging to acquire functional regeneration of native enthesis at the tendon-to-bone interface.

The poor healing response following rotator cuff tendon injuries is multifactorial but likely related to inflammation and extracellular matrix (ECM) disturbance 9. Additionally, it is widely accepted that insufficient and disorganized expression of cytokines hinders tendon-to-bone interface healing 10. Previous studies showed that inflammatory cells at the tendon-to-bone interface precipitate scar formation, that bony ingrowth into the tendon from the prepared tuberosity is slow and limited, and that poor response is exacerbated by an insufficient number of undifferentiated stem cells 11. Additionally, oxidative stress is involved in tendon repair after injury and plays a pathological role in rotator cuff tears 12. Inflammatory cytokines have been demonstrated to play a key role in oxidative stress-induced cellular apoptosis, which is mediated by activation of caspase 13. Furthermore, studies have reported that the balance between matrix metalloproteinases (MMPs) and their inhibitors is disrupted in rotator cuff tears 14, 15. These problems highlight the urgent need for an intelligent delivery system that can provide precisely controlled delivery of anti-inflammatory drugs *in vivo*. For example, curcumin has shown powerful antioxidative and anti-inflammatory effects and is beneficial for cartilage regeneration 16. However, its therapeutic efficacy is restricted by its relatively poor oral bioavailability 17. Therefore, we speculate that precise *in situ* release of curcumin might be a promising strategy to improve the healing response of rotator cuff tears.

Additionally, it is generally believed that a lack of endogenous stem cells at the wound site contributes to failure of rotator cuff healing 6. Magnesium alloys have attracted great attention in the research and development of biomaterials for clinical applications due to their bioactive and biodegradable characteristics 18, 19. Magnesium ion (Mg^2+^) has been reported to effectively enhance stem cell aggregation and promote fibrocartilage regeneration 20, 21. Moreover, our prior work demonstrated that Mg^2+^ effectively improved regeneration of the fibrocartilage layer at the tendon-to-bone interface in a rabbit rotator cuff tear model 22.

**Figure 1:**
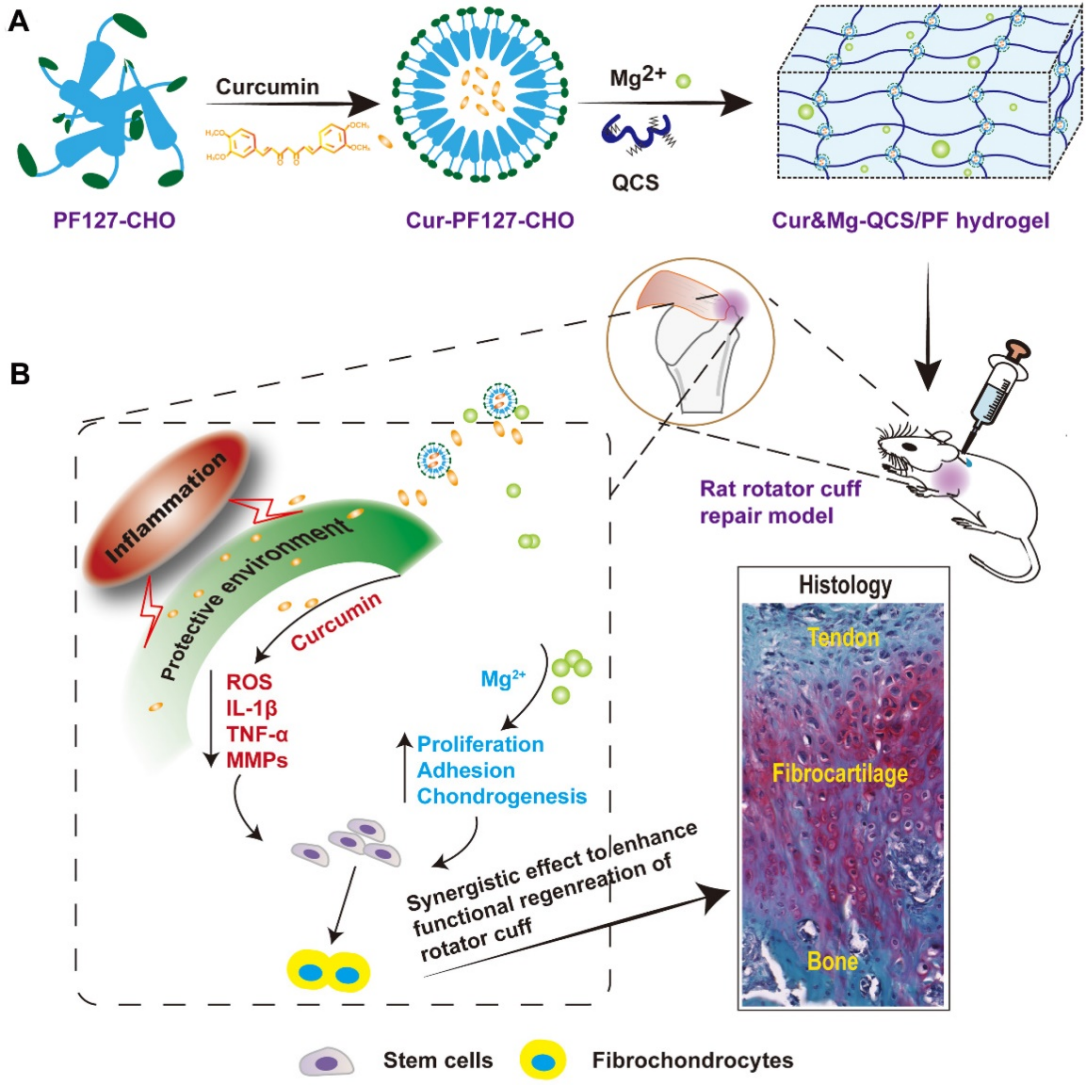
(A) Synthesis scheme of Cur&Mg-QCS/PF. (B) Injection of Cur&Mg-QCS/PF mediating simultaneous *in situ* release of curcumin and Mg^2+^. The released curcumin provided a protective environment for stem cells at the repair site via anti-inflammatory and antioxidative effects. In addition, the released Mg^2+^ enhanced stem cell adhesion, proliferation, and chondrogenesis during rotator cuff healing. In conclusion, the composite hydrogel significantly improved regeneration of the fibrocartilage interface and integration of tendon and bone via the synergistic effects of the two factors.

Based on the involvement of multiple factors in the pathogenesis of rotator cuff tears, we hypothesized that a combination therapy targeting multiple factors might be superior to monotherapy. In particular, curcumin can protect cells near the tendon-to-bone interface from apoptosis and the tendon matrix from degradation by inhibiting inflammatory factors and reactive oxygen species (ROS), while Mg^2+^ can enhance healing of the tendon-to-bone interface. However, sustained release of curcumin and Mg^2+^ from a delivery system has not been reported. In this study, we developed a composite self-healing hydrogel to provide precisely controlled delivery of curcumin and Mg^2+^ simultaneously, and demonstrated that it synergistically enhanced the *in vivo* healing of rotator cuff tears. We previously formed hydrogels through dynamic Schiff base bonding between amino groups from quaternized chitosan (QCS) and aldehyde groups from benzaldehyde-terminated Pluronic®F127 (PF127-CHO) polymer 23. The QCS/PF hydrogel exhibited excellent injectability, rapid self-healing, and adhesive properties, which should enable easy injection and consequent adaptation to the irregularly shaped supraspinatus insertion site. Curcumin was loaded in the PF127-CHO micelles, which greatly enhanced its solubility. In this study, we also loaded Mg^2+^ into QCS/PF through complexation between Mg^2+^ and -NH_2_/NH, -OH in the QCS main chain following our previous study 22. The Cur&Mg-QCS/PF hydrogel provided a protective environment for stem cells during rotator cuff healing through the anti-inflammatory and antioxidative effects of the released curcumin (Figure 1). Additionally, the composite hydrogel released Mg^2+^ to enhance stem cell migration, adhesion, proliferation, and chondrogenesis at the injury site (Figure 1), providing a two-pronged approach to enhance tendon-to-bone healing. Moreover, *in vivo* experiments suggested that the synergistic effects of curcumin and Mg^2+^ could significantly promote functional healing of the tendon-to-bone interface. This is the first study providing proof of concept for Cur&Mg-QCS/PF as an effective drug delivery platform to mediate simultaneous release of curcumin and Mg^2+^ to promote rotator cuff healing.

## Methods

### Materials

Chitosan (M_n_ = 100,000-300,000 Da) was obtained from J&K Scientific Ltd. Glycidyltrimethylammonium chloride, Pluronic® F127 (PF127), 4-hydroxybenzaldehyde, MgCl_2_•6H_2_O, and curcumin were purchased from Sigma-Aldrich. All other reagents were used without further purification.

### Synthesis of Cur-QCS/PF, Mg-QCS/PF, and Cur&Mg-QCS/PF

QCS and PF127-CHO were synthesized based on our previous work 23. QCS polymer was dissolved in phosphate-buffered saline (PBS) at 55 °C to form a 5% (w/v) solution. MgCl_2_•6H_2_O was added to PF127-CHO or curcumin-encapsulated PF127-CHO (Cur-PF127-CHO) solution. The hydrogels were prepared by mixing QCS solution and PF127-CHO/Cur-PF127-CHO solution with or without MgCl_2_•6H_2_O at a ratio of 2:3 (v/v) at 37 °C.

### Characterization of the hydrogels

The hydrogels were evaluated by Fourier transform infrared spectroscopy (FT-IR) 23 and their morphology 24-26, injectability 27, adhesive ability 28, 29, self-healing performance 30, 31, rheological properties 32, 33, and *in vitro* and *in vivo* degradation profiles 28 were tested. The details are available in the supplementary information.

### *In vitro* drug release assay

The release profiles of curcumin and Mg^2+^ from the hydrogels were studied following reported methods 23, 34. The details are available in the supplementary information.

### Biocompatibility assays

The cytocompatibility of the hydrogels was investigated by a direct contact test between rat bone marrow stromal cells (BMSCs) and hydrogel based on reported methods 35, 36. The details are available in the supplementary information.

Additionally, the histocompatibility of the hydrogels was investigated by subcutaneous implants based on our previous work 35. Briefly, bulk hydrogel was implanted under the skin of rats, and then tissue samples were taken 1, 2, and 4 weeks after surgery for H&E staining and inflammation evaluation.

### *In vitro* apoptosis assay

To investigate the anti-apoptotic effects of the hydrogels on rat BMSCs, hydrogen peroxide (H_2_O_2_) was used as an inducer. Rat BMSCs were seeded on cell culture plates at a density of 0.5 or 1×10^5^ cells/mL. Then, the cells were pretreated with curcumin-doped hydrogel extracts for 2 h and then exposed to H_2_O_2_ (at a final concentration of 100 μM) for 10 h. Subsequently, Cell Counting Kit-8 (CCK-8), Hoechst 33258 staining, and H2DCF-DA assay were performed to evaluate cell viability, apoptosis, and intracellular ROS according to the manufacturers' instructions.

### Rat model of acute rotator cuff tear and repair

Based on our prior work 22, male Sprague Dawley rats (230-250 g) were used to construct a rotator cuff tear and repair model with the hydrogels injected into the tendon-to-bone interface. Briefly, rats were anesthetized, their skin was prepared, and a longitudinal incision was made over the shoulder joint. Subsequently, the supraspinatus tendon was sharply cut and a bone grove was created at the greater tuberosity. Hydrogels (50 μL) were injected into the bone grove and then the retracted tendon stump was pulled to cover the groove and fixed with a 6-0 non-absorbable surgical suture. In blank rats, the retracted tendon was pulled to cover the bone grove without any hydrogel injection. The wound was closed and the animals were given free access to water and food in the cage. Additional details are provided in the supplementary information. Animal experiments were approved by animal research committee of Xi'an Jiaotong University.

### *In vivo* assessment of the protective effects of Cur-QCS/PF on repaired tendon

The protective effects of Cur-QCS/PF on repaired tendon were evaluated in the rotator cuff tear model described above with some modifications. Briefly, after the supraspinatus tendon was sharply cut, the retracted tendon stump was directly pulled to cover the bone surface without artificial groove and fixed with a 6-0 non-absorbable surgical suture and then hydrogels (50 μL) were injected into the tendon and bone junction site. The animals were allocated into two groups: blank and Cur-QCS/PF. Rats were sacrificed 3, 14, and 21 days after surgery. Tendon repair was evaluated by histology. Subsequently, the underlying mechanism was investigated by immunofluorescence assays using standard protocols.

### *In vitro* adhesion and chemotaxis assays

The adhesion and chemotaxis effects of the hydrogels on rat BMSCs were investigated following previously reported procedures with some modifications 20, 22, 37. The details are available in the supplementary information.

### *In vivo* stem cell aggregation assay

Immunofluorescence staining was used to investigate the recruitment of stem cells *in vivo*. A rat model of acute rotator cuff tear and repair was generated following the above method and injected with Mg-QCS/PF or Cur&Mg-QCS/PF or left untreated. Samples of the tendon-humerus complex were harvested 2 weeks after surgery for immunofluorescence analysis. The sections were stained with FITC anti-rat CD44 antibody to detect mesenchymal stem cells (MSCs). The nuclei were counterstained with DAPI. Slides were observed under an inverted fluorescence microscope. The CD44-positive area was quantified using NIH ImageJ software.

### *In vitro* fibrochondrogenic differentiation assay

For analysis of fibrocartilage markers (COL2A1, SOX-9, ACAN, Decorin), 2×10^4^ rat BMSCs were seeded on 6-well plates and co-cultured with hydrogel disks. After 7 and 14 days of culture, the gene expressions of fibrocartilage markers and BMP-2 were determined by RT-qPCR according to our previous study 22. The details are available in the supplementary information and the primers are listed in Table S1.

### *In vivo* assessment of tendon-to-bone healing

The rat model of acute rotator cuff tear and repair described above was used to evaluate the positive effects of the composite hydrogel on tendon-to-bone healing. Rats were randomly divided into 3 groups and injected with QCS/PF or Cur&Mg-QCS/PF at the tendon-to-bone interface or left untreated. Histology, immunofluorescence assays, and biomechanical tests were used to evaluate postoperative tendon-to-bone healing. The details are available in the supplementary information.

### Statistical analysis

Statistical analysis was conducted using IBM SPSS v25.0. For quantitative data, independent samples t-test and one-way ANOVA were used for statistical analysis, and least significant difference test was used for post hoc analysis. *P* < 0.05 was considered statistically significant.

## Results and Discussion

### Synthesis of the hydrogels

**Figure 2:**
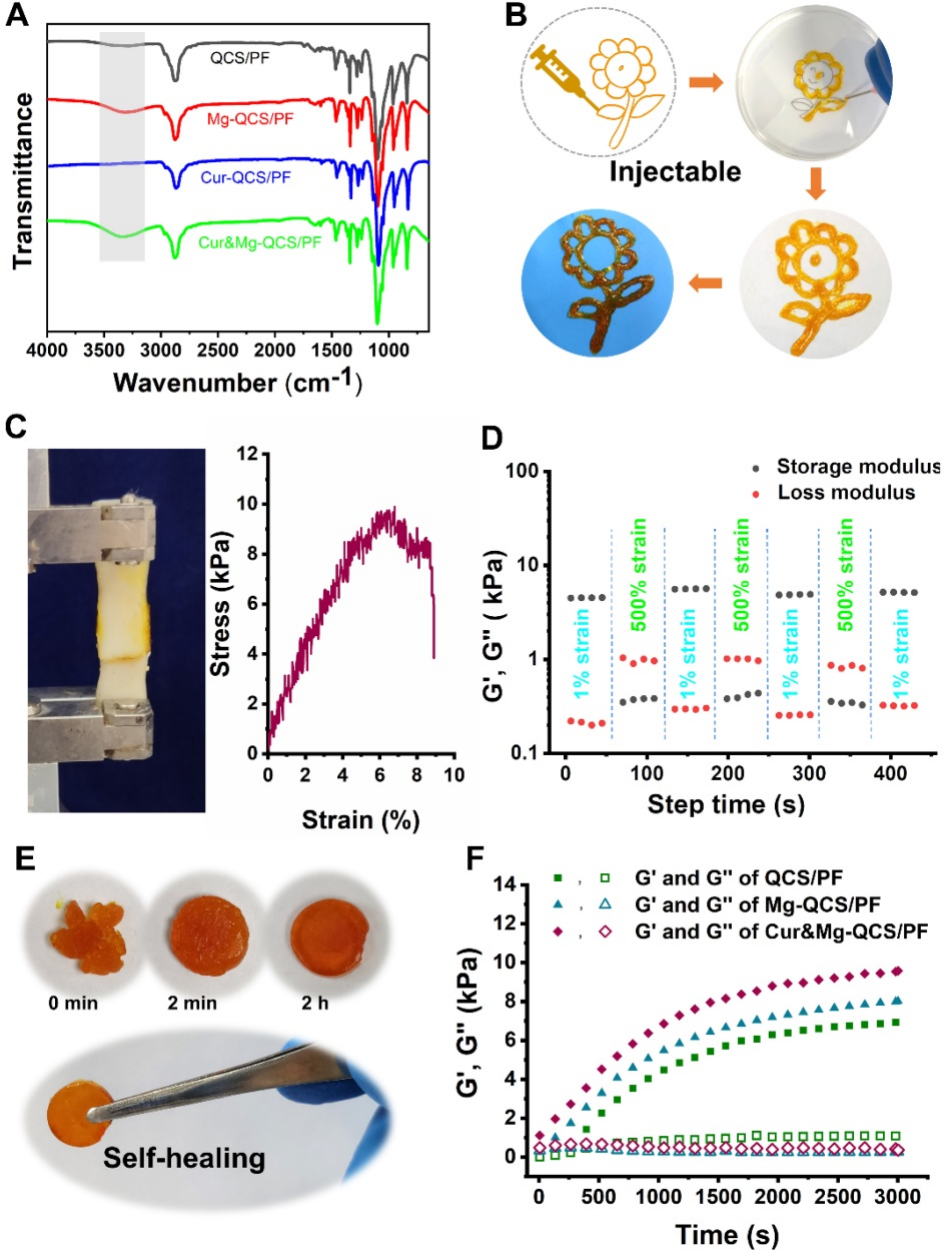
(A) FT-IR spectra of QCS/PF, Mg-QCS/PF, Cur-QCS/PF, and Cur&Mg-QCS/PF. (B) Injectability of Cur&Mg-QCS/PF. (C) Adhesiveness of Cur&Mg-QCS/PF to porcine skin. (D) Quantitative self-healing tests of Cur&Mg-QCS/PF. (E) Macroscopic self-healing tests of Cur&Mg-QCS/PF. (F) Rheological performance of the hydrogels.

To provide a suitable microenvironment for tendon-to-bone healing during rotator cuff repair, we designed an injectable composite hydrogel with excellent anti-inflammatory performance and the simultaneous ability to promote stem cell chondrogenesis. We constructed a novel active drug delivery vector based on QCS/PF, and successfully overcame the challenge of simultaneous high-efficiency loading and controlled release of Mg^2+^ and curcumin. Based on metal coordination between Mg^2+^ and QCS 22, 38 and the solubilization effect of PF127-CHO nanomicelles on hydrophobic curcumin 23, the two drugs were stably encapsulated into QCS/PF (Figure S1) and were slowly released during the gradual degradation of the hydrogel. After mixing Mg^2+^, PF127-CHO/Cur-PF127-CHO, and QCS together, hydrogels were formed based on the dynamic Schiff base bond between -NH_2_ from QCS and -CHO from PF127-CHO 23, which was confirmed by FT-IR in Figure S2. Compared with QCS/PF and Cur-QCS/PF without Mg^2+^ addition, Mg-QCS/PF and Cur&Mg-QCS/PF exhibited wide IR peaks in the range of 3200-3500 cm^-1^, confirming the coordination of Mg^2+^ and QCS (Figure 2A) 39, 40. According to our previous work 22, 23, hydrogels with QCS and PF127 concentrations of 2% and 18% (w/w) were selected for Mg^2+^ and curcumin encapsulation. The hydrogels were named QCS/PF, Mg-QCS/PF (QCS/PF loaded with Mg^2+^), Cur-QCS/PF (QCS/PF loaded with curcumin), and Cur&Mg-QCS/PF (QCS/PF loaded with Mg^2+^ and curcumin).

### Characterization of the hydrogels

The composite hydrogel exhibited excellent injectability, adhesiveness, and self-healing. Owing to its shear-thinning performance 41, Cur&Mg-QCS/PF could be deposited with a syringe to form a gel with the shape of a sunflower, which retained this shape when stripped from the original interface (Figure 2B). Injectability is crucial for biomedical applications of hydrogels, especially for easy application in the irregularly shaped supraspinatus insertion site 22, 42. Additionally, compared with traditional uninjectable scaffolds, injectable hydrogels can freely fill irregular lacuna between rotator cuff tendon and bone, thereby better promoting regeneration of the tendon-to-bone interface. Figure 2C presents results from a lap shear test of the composite hydrogel. Adhesion of the hydrogel to pig skin reached nearly 10 kPa, which is an ideal strength for the hydrogel to adhere to the rotator cuff without shedding or shifting with tissue activity. Additionally, the composite hydrogel exhibited good self-healing performance (Figure 2D-E). Furthermore, the rheological recovery test results in Figure 2D further confirmed that the healed hydrogel had the same modulus as the original gel. Self-healing can prolong the lifespan of the hydrogel network under mechanical force, especially in the tendon-to-bone interface 43. Moreover, a self-healing vehicle would improve the safety of the hydrogel during the rotator cuff repair process.

The morphologies of the hydrogels were investigated by scanning electron microscopy (SEM). As shown in Figure S3, all the hydrogels exhibited a highly porous structure. After curcumin and Mg^2+^ were entrapped into the hydrogel network, a decrease in pore size was observed (Figure S3). This decrease in pore size can be attributed to the denser cross-linking network from Mg^2+^ and curcumin addition. Hydrogel pore morphology is of utmost importance in tissue engineering. A highly porous structure facilitates exchange of oxygen, nutrients, and metabolic waste. Therefore, with its porous structure, Cur&Mg-QCS/PF has the potential to promote rotator cuff healing.

The storage modulus (G′) and loss modulus (G″) of the hydrogels were measured to evaluate the rheological performance of the hydrogels. As shown in Figure 2F, Cur&Mg-QCS/PF and Mg-QCS/PF exhibited higher G′ than QCS/PF. Additionally, with the addition of curcumin, the Cur&Mg-QCS/PF networks were stronger than those of Mg-QCS/PF. Due to the special biomechanical environment of the rotator cuff tendon-to-bone junction site 44, mechanical properties must also be considered when developing a tissue engineering hydrogel scaffold. The mechanical performance of the hydrogels was determined using a cyclic compression test. As shown in Figure S4, all the hydrogels remained intact under 60% strain after 20 loading cycles, indicating that the hydrogels have good robustness and resilience. Moreover, under the same strain, the stress of Cur&Mg-QCS/PF was higher than that of Mg-QCS/PF and QCS/PF, indicating the positive effect of Mg^2+^ and curcumin on hydrogel strength (Figure S4). Therefore, the addition of curcumin and Mg^2+^ improved the mechanical properties of the hydrogel network, which endowed the hydrogels with the ability to structurally support tissue in-growth while resisting *in vivo* compressive stress between rotator cuff tendons and bone. In conclusion, these results suggested that Cur&Mg-QCS/PF had a suitable and well-maintained network, which should enable sustained release of Mg^2+^ and curcumin simultaneously.

### Degradation and Mg^2+^/curcumin release kinetics of the hydrogels

**Figure 3:**
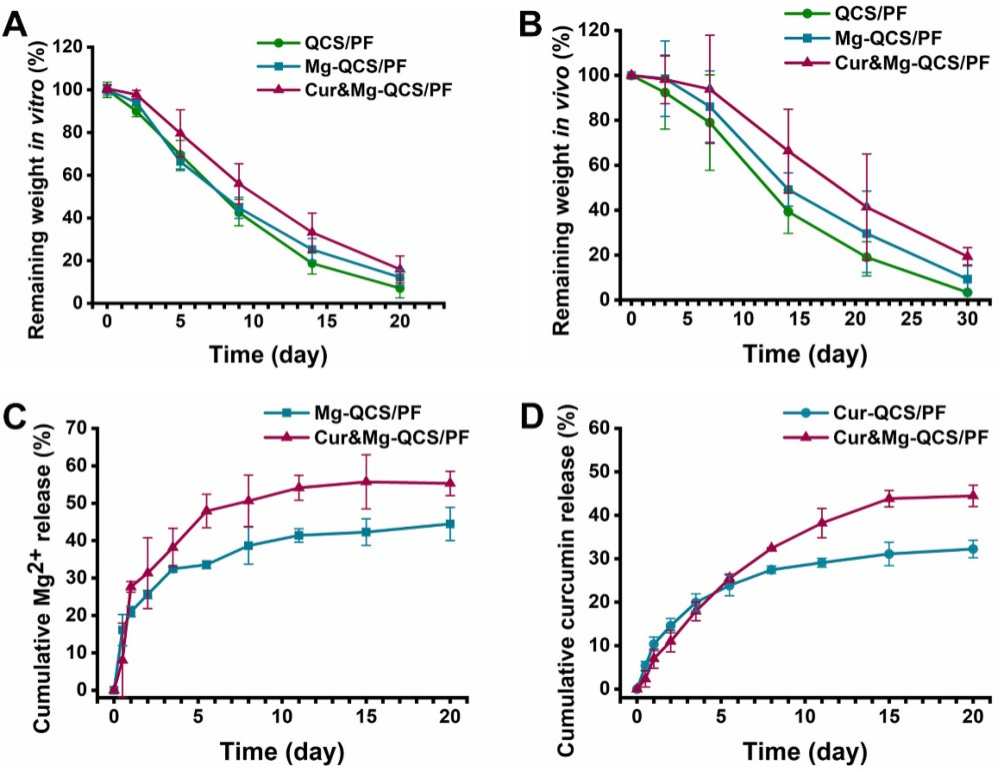
(A) Degradation profiles of the hydrogels *in vitro* and (B) *in vivo*. (C) Cumulative release of Mg^2+^ from Mg-QCS/PF and Cur&Mg-QCS/PF in PBS. (D) Cumulative release of curcumin from Cur-QCS/PF and Cur&Mg-QCS/PF in PBS.

Suitable hydrogel degradation performance is also essential for tissue regeneration 45. As shown in Figure 3A, all three hydrogels exhibited >20% mass remaining after 15 days incubation in PBS. Notably, with the addition of Mg^2+^ and curcumin, the hydrogels showed slower degradation, which may be related to the denser internal cross-linking network of the hydrogels. In addition, the *in vivo* degradation behavior of the hydrogels was investigated, and the results exhibited similar trends as the* in vitro* degradation behaviors (Figure 3B). The degradation behavior of Cur&Mg-QCS/PF could be beneficial for tissue regeneration 46. Degradation of the hydrogel network is conducive to tissue in-growth and extracellular matrix formation, which are critical to rotator cuff healing.

Controlled and precise release of therapeutic agents is particularly advantageous in tissue engineering applications that require the use of bioactive cues to create a suitable microenvironment and direct cell-matrix interactions 46. Simultaneous loading of multiple bioactive molecules within the hydrogel network to obtain sustained release enhances regulation of cell behaviors and extracellular matrix biosynthesis. However, owing to the intrinsic permeability of hydrogels and limited network interactions with bioactive molecules, highly efficient loading and simultaneous delivery of diverse drugs with different properties is challenging 47. Our previous work demonstrated that QCS/PF exhibits suitable curcumin 23 and Mg^2+^ 22 release. However, it is not clear whether curcumin and Mg^2+^ will affect each other's release behavior when the two drugs are encapsulated in the same hydrogel. Herein, the release kinetics of Mg^2+^ and curcumin from the hydrogels were investigated. As shown in Figure 3C, >40% of Mg^2+^ remained in Cur&Mg-QCS/PF after 20 days of incubation in PBS and the cumulative released Mg^2+^ from Cur&Mg-QCS/PF was higher than that from Mg-QCS/PF. These results indicated that Cur&Mg-QCS/PF could be a suitable delivery vehicle for Mg^2+^ in rotator cuff healing. The release profile of curcumin from the hydrogels was also investigated *in vitro*. Compared with Cur-QCS/PF, Cur&Mg-QCS/PF exhibited slower initial release of curcumin but higher cumulative release (Figure 3D). In summary, Cur&Mg-QCS/PF has the potential to deliver sustained release of Mg^2+^ and curcumin simultaneously for rotator cuff healing.

### Biocompatibility of the hydrogels

Good biocompatibility is imperative for tissue engineering applications of biomedical materials 23. Therefore, *in vitro* and *in vivo* tests were performed to investigate the biocompatibility of the hydrogels. First, CCK-8 and LIVE/DEAD assays were performed to examine the *in vitro* biocompatibility of Cur&Mg-QCS/PF. As shown in Figure S5A-B, the hydrogels group exhibited excellent cell compatibility over 5 days of incubation. BMSCs treated with Cur&Mg-QCS/PF or Mg-QCS/PF showed increased proliferation compared to cells treated with hydrogels without Mg^2+^ (Figure S5B).These results are consistent with previous reports that demonstrated the capability of Mg^2+^ to promote BMSCs growth 21. Second, a subcutaneous implantation experiment was performed to investigate the histocompatibility of the hydrogels. As shown in Figure S5C, slight inflammation was detected 1 week after implantation, which nearly disappeared by 4 weeks after implantation. All three hydrogels exhibited significant degradation at 4 weeks after implantation. Furthermore, vital organs including heart, liver, spleen, lung, and kidney were harvested and stained by H&E. There was no significant histological difference between the hydrogel and blank groups (Figure S5D), indicating the good histocompatibility of the hydrogels. In conclusion, the hydrogels exhibited good biocompatibility for application to rotator cuff healing.

### Anti-inflammatory effects of curcumin-loaded hydrogels

**Figure 4:**
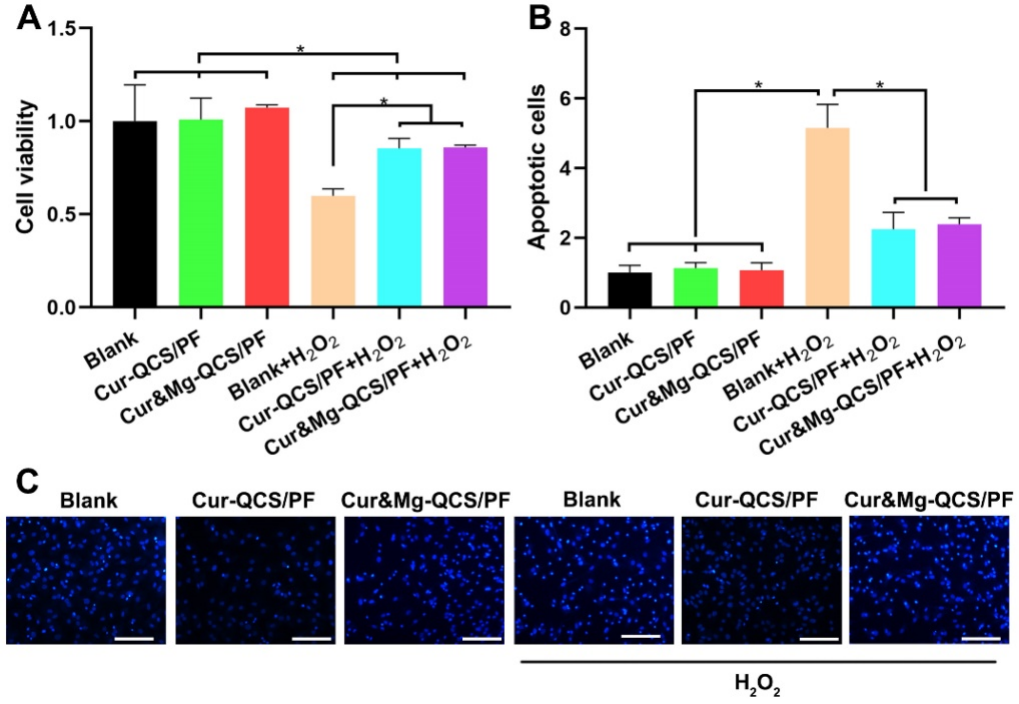
*In vitro* anti-apoptosis and antioxidant effects of curcumin-loaded hydrogels. (A) Cell viability and (B) apoptosis rate of rat BMSCs exposed to H_2_O_2_ with and without treatment with curcumin-loaded hydrogels (n = 3). The data were normalized to the no treatment group. *P < 0.05. (C) Representative images of apoptotic cells stained with Hoechst 33258. Scale bar: 100 µm.

MSCs have been accepted as cell sources with the potential to be an effective strategy for rotator cuff healing 48. However, cells around the injury site are confronted with apoptosis under the difficult conditions of inflammatory infiltration and enormous free radicals 49. In this study, oxidative injury was induced by exposing rat BMSCs to H_2_O_2_, and the protective effect of curcumin released from hydrogels was evaluated using a CCK-8 assay. As shown in Figure 4A, the cell viability of rat BMSCs decreased significantly after exposure to H_2_O_2_. However, higher cell viability was measured in H_2_O_2_-exposed cells treated with Cur-QCS/PF or Cur&Mg-QCS/PF compared with no treatment. These results indicated that curcumin released from the hydrogels inhibited damage to BMSCs from H_2_O_2_ exposure. Consistent with these results, fewer cells were stained with bright blue fluorescence (Hoechst 33258) in the Cur-QCS/PF and Cur&Mg-QCS/PF groups than the no treatment group (Figure 4B-C), indicating that curcumin reduced the number of apoptotic cells and nuclear condensation. Additionally, the intracellular ROS levels were investigated by H2DCF-DA staining. As shown in Figure S6, the released curcumin significantly reduced the production of intracellular ROS. Taken together, these results suggested that curcumin-encapsulated hydrogels protected BMSCs from H_2_O_2_-induced apoptosis, indicating their potential for promoting healing of the tendon-to-bone interface.

**Figure 5:**
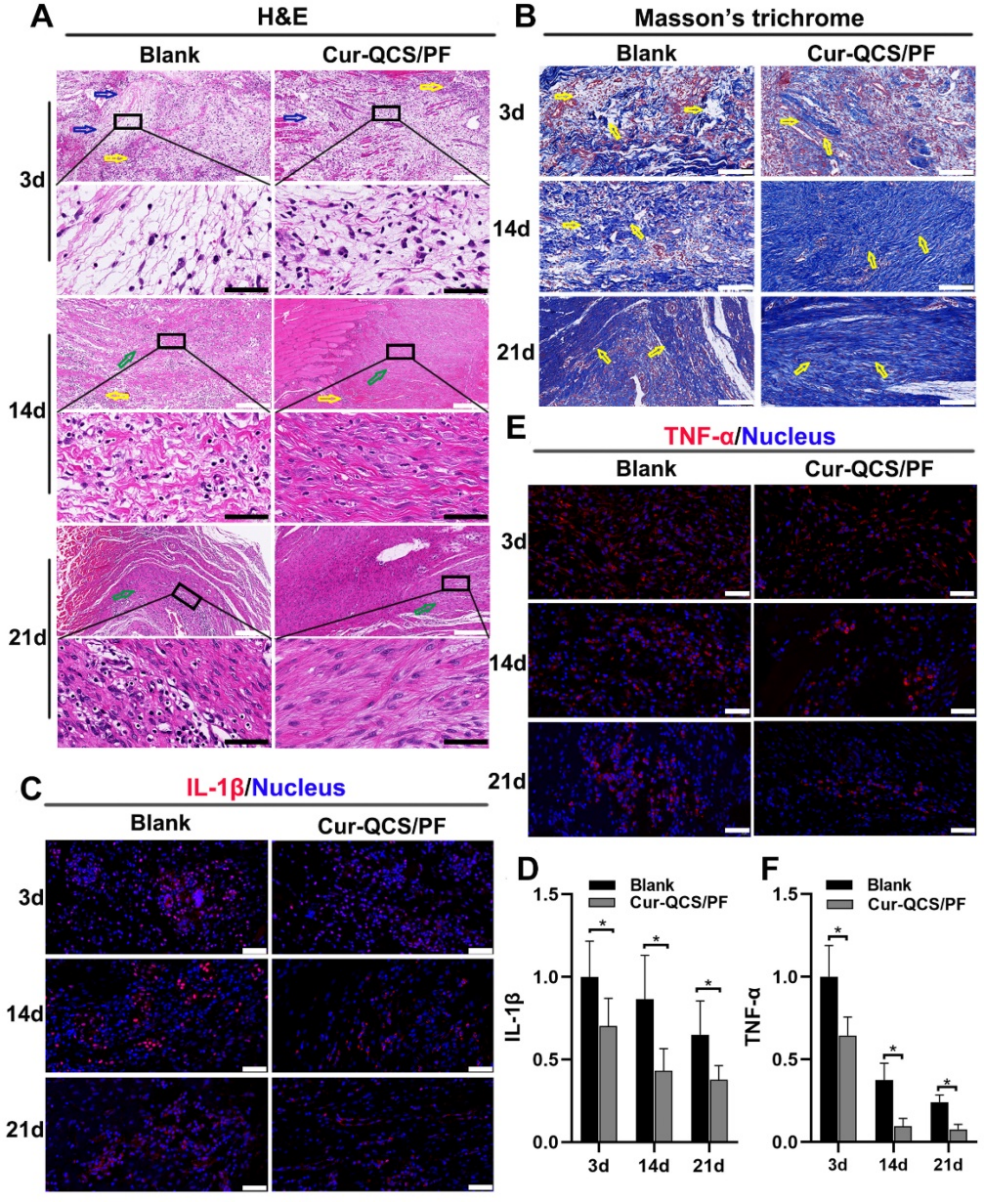
*In vivo* anti-inflammatory protective effects of curcumin-loaded hydrogels. (A) Representative images of repaired tendon stained with H&E. The yellow arrows indicate inflammatory cells, the blue arrows indicate tendon ECM degradation and disorganization, and the green arrows indicate tendon fiber. White scale bars: 200 µm, black scale bars: 60 µm. (B) Representative images of repaired tendon stained with Masson's trichrome. The yellow arrows indicate tendon fiber. Scale bars: 100 µm. (C) Representative images of repaired tendon stained for IL-1β. Scale bars: 50 µm. (D) Relative expression of IL-1β in tendon tissue (n = 6). (E) Representative images of repaired tendon stained for TNF-α. Scale bars: 50 µm. (F) Relative expression of TNF-α in tendon tissue (n = 6). The data were normalized to the blank group on day 3. *P < 0.05.

Sustained tendon matrix disorganization with inflammation has been recognized as a major cause of pain and dysfunction in rotator cuff tendon injuries 50. Upregulation of inflammatory cytokines and increased oxidative stress have been tightly associated with rotator cuff tear pathology and impede healing responses 51. These cytokines are believed to be involved in the maintenance of tendon matrisome homeostasis by regulating the expression of extracellular matrix genes, especially type I collagen by tenoblasts and tenocytes 9. Figure 5A shows a significant change in the architecture, morphology, and ECM organization of repaired rotator cuff tendon with different treatments. ECM disorganization was high at 3 days after surgery, and disorganized collagen fibers were detected at 14 days in the blank group, but ECM organization was significantly better in the Cur-QCS/PF group. Additionally, inflammation in the rotator cuff tendon was evident by the increased number of leukocytes. However, the tendon tissues in the blank group exhibited more flaky vacuolar structures, which might be the result of cell necrosis and absorption, than the Cur-QCS/PF group, suggesting more serious matrix degradation. Consistent with this result, Masson's trichrome staining revealed significant disorganization of collagen fibers in the rotator cuff tendons of the blank group compared with the Cur-QCS/FP group (Figure 5B). The major alterations in ECM composition in the rotator cuff tendon insertion site after surgery were consistent with those found by H&E staining. Notably, the QCS/PF and blank groups showed similar histological characteristics at corresponding timepoints after surgery without significant differences in tendon protection (Figure S7A-B). These results indicated that curcumin released from the hydrogels inhibited inflammation and degradation of ECM in tendon tissues, which are associated with a beneficial environment for tendon repair after injury.

**Figure 6:**
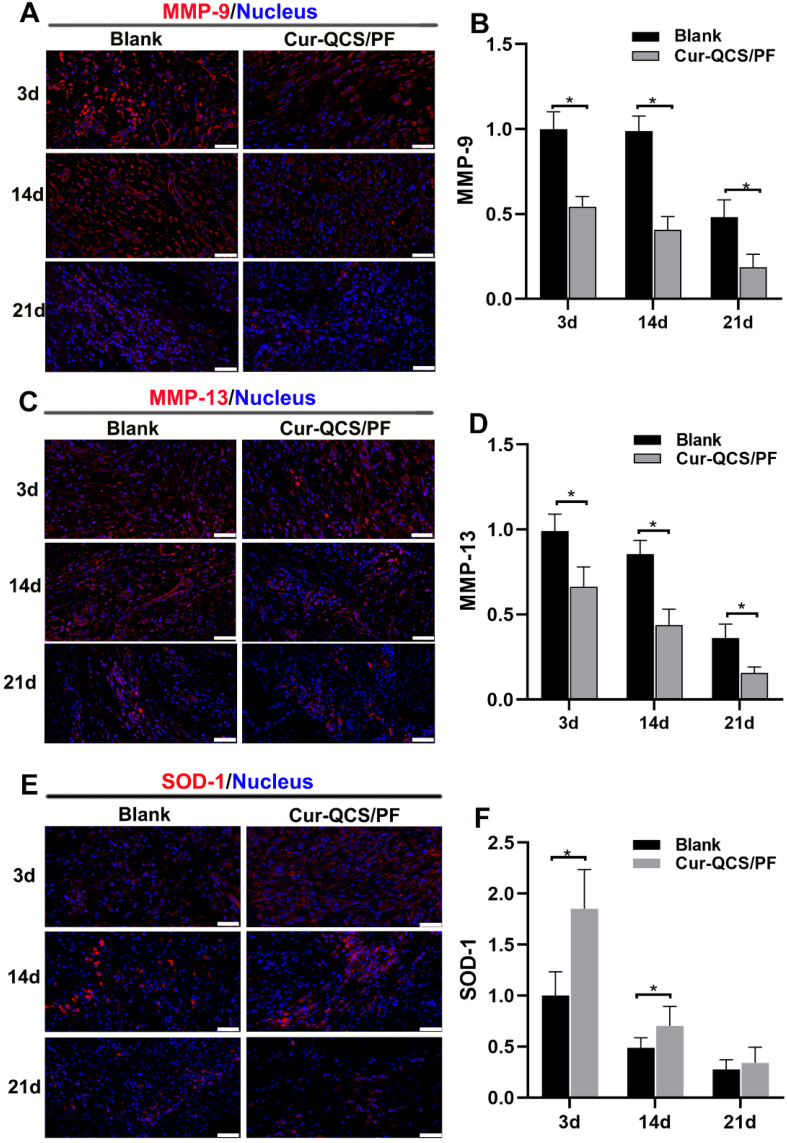
*In vivo* protective effects of curcumin-loaded hydrogels on matrix degradation and oxidation. A) Representative images of repaired tendon stained for MMP-9. Scale bars: 50 µm. (B) Relative expression of MMP-9 in tendon tissue (n = 6). (C) Representative images of repaired tendon stained for MMP-13. Scale bars: 50 µm. (D) Relative expression of MMP-13 in tendon tissue (n = 6). (E) Representative images of repaired tendon stained for SOD-1. Scale bars: 50 µm. (F) Relative expression of SOD-1 in tendon tissue (n = 6). The data were normalized to the blank group on day 3. *P < 0.05.

In order to investigate the potential mechanism of curcumin-loaded hydrogel inhibition of tendon matrix degradation, the expressions of inflammatory cytokines were detected by immunofluorescence staining. As shown in Figure S7C-F, in the early phase of rotator cuff healing after injury, the blank and QCS/PF groups showed increased interleukin-1β (IL-1β) and tumor necrosis factor alpha (TNF-α) expression. This trend continued for at least 14 days. In comparison, the Cur-QCS/PF group showed significantly lower expression of IL-1β and TNF-α during the healing process (Figure 5C-F). This difference in the activities of inflammatory cytokines was consistent with histology results. IL-1β is well known to initiate a cascade of catabolic responses by upregulating a group of degradative enzymes including MMP-1, MMP-9, and MMP-13 52. Shindle et al. reported that increased joint inflammation is closely correlated with tendon remodeling, and the authors found strong correlations between IL-1β-related cytokines and tissue remodeling genes (MMP-9 and MMP-13) in the torn supraspinatus tendon 10. Increased pro-inflammatory mediators like IL-1β and TNF-α act as a signal for MMP overexpression, resulting in ECM degradation and rotator cuff tear 10. Therefore, we investigated the expressions and distributions of MMP-9 and MMP-13 in repaired tendon tissues. Similar to IL-1β and TNF-α, MMP-9 and MMP-13 were more highly expressed in the QCS/PF and blank groups than the Cur-QCS/PF group (Figure 6A-D and Figure S8A-D). MMPs are critical to the dynamic homeostasis of the extracellular matrix 11, 53. Previous work has demonstrated that increased MMP activity is closely related to degenerative tendinopathy and rotator cuff pathology 14, 54. Bedi et al. reported that inhibition of MMP expression in the rotator cuff insertion site significantly improved the formation of fibrocartilage and reduced collagen degradation 55, 56. In our study, immunofluorescence staining results suggested that Cur-QCS/PF inhibited overexpression of inflammatory cytokines to protect the rotator cuff tendon matrix from degradation via *in situ* release of curcumin. Interestingly, higher superoxide dismutase-1 (SOD-1) protein expression was observed in the repaired tendon tissues of the Cur-QCS/PF group compared with the blank group (Figure 6E-F), indicating its potential to improve antioxidant expression. Additionally, there was no significant difference in SOD-1 expression between the QCS/PF and blank groups (Figure S8E-F). Oxidative stress resulting from an imbalance between oxidation caused by ROS and reduction catalyzed by antioxidant systems was reported to be closely involved in tendon regeneration 12. SOD-1 is a typical antioxidant enzyme that scavenges ROS *in vivo* 57. Previous work reported that rotator cuff regeneration was more easily induced by excessive oxidative stress in SOD-1‐deficient mice than normal mice 57, 58. Therefore, considering the role of oxidative stress in rotator cuff degeneration and repair, *in situ* delivery of antioxidants such as curcumin is beneficial for tendon-to-bone healing. Overall, these results suggested that curcumin-loaded hydrogels effectively inhibited the inflammatory response, scavenged free radicals, increased the expression of antioxidants, significantly reduced BMSCs apoptosis and tendon matrix degradation, and provide a protective microenvironment for tendon-to-bone healing.

Pro-inflammatory cytokines are closely related to the pathological process of rotator cuff tears. Therefore, inhibiting excessive inflammation might reduce the progress of tendon tears and improve clinical healing, which is of great significance for the treatment of rotator cuff injury. However, current studies on tissue engineering repair of rotator cuff injury are mostly limited to the provision of growth factors and scaffolds, and there are few reports on measures to improve the inflammatory microenvironment after rotator cuff injury. Our study demonstrates the positive significance of an anti-inflammatory strategy for rotator cuff repair, and provides a promising therapeutic measure for rotator cuff healing.

### Pro-chondrogenesis effects of Mg^2+^-loaded hydrogels

**Figure 7:**
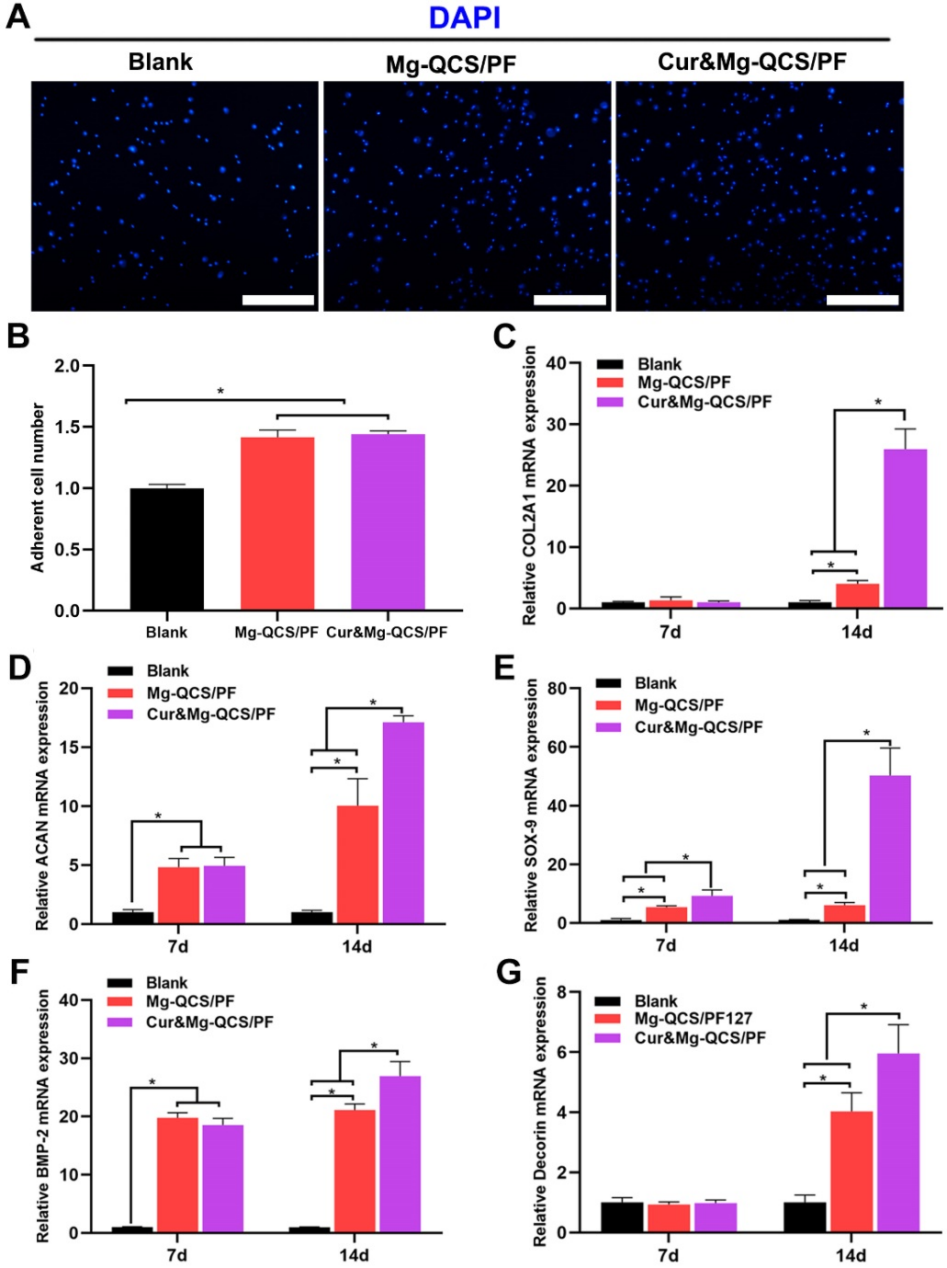
*In vitro* cellular adhesion and fibrocartilage differentiation of BMSCs co-cultured with Mg^2+^-loaded hydrogels. (A) Representative images of adherent BMSCs stained with DAPI. Scale bars: 100 µm. (B) Number of adherent cells (n = 3). (C) Relative COL2A1 gene expression (n = 3). (D) Relative ACAN gene expression (n = 3). (E) Relative SOX-9 gene expression (n = 3). (F) Relative BMP-2 gene expression (n = 3). (G) Relative decorin gene expression (n = 3). The data were normalized to the blank group. *P < 0.05.

Mg^2+^ has been reported to improve the expression of fibronectin and integrin at cell adhesion sites, which are essential to cell spreading and generation of cellular tension 37. As shown in Figure 7A-B, Mg-QCS/PF and Cur&Mg-QCS/PF significantly enhanced BMSC adhesion compared to no treatment. The addition of curcumin into the hydrogels did not affect cell adhesion compared with Mg-QCS/PF. Therefore, sustained release of Mg^2+^ from Mg-QCS/PF and Cur&Mg-QCS/PF promoted cellular adhesion and interaction with the hydrogel network, thereby facilitating spreading and accumulation of the stem cells.

To further investigate the effects of the two drugs on cell behavior, BMSCs were co-cultured with hydrogel disks in chondrogenic medium. After 7 or 14 days of culture, the majority of BMSCs in all groups remained viable, further indicating that all of the hydrogels were cytocompatible. As shown in Figure 7D-E, higher mRNA expressions of ACAN and SOX-9 were detected in the Mg-QCS/PF and Cur&Mg-QCS/PF groups than the blank group on day 7. By day 14, the mRNA expressions of COL2A1, ACAN, SOX-9, and decorin significantly increased in both the Mg-QCS/PF and Cur&Mg-QCS/PF groups compared to the blank group (Figure 7C-E and G). Interestingly, the Cur&Mg-QCS/PF group exhibited higher expression of fibrocartilage markers than the Mg-QCS/PF group on day 14, probably because curcumin enhanced the ability of Mg^2+^ to promote the expression of fibrocartilage markers. This result agreed with previous work by Kim et al., who reported that adding an appropriate amount of curcumin to a silk scaffold improved chondrocyte proliferation and chondrogenesis for cartilage repair 16. Furthermore, higher BMP-2 mRNA expression was detected in the Mg-QCS/PF and Cur&Mg-QCS/PF groups on day 7 (Figure 7F), indicating that Mg^2+^ released from the hydrogels effectively stimulated BMP-2 expression, which has been reported to play an important role in chondrogenesis 21. In summary, incorporation of both curcumin and Mg^2+^ into the QCS/PF hydrogels not only improved the mechanical performance of the hydrogel, but also provided synergistic antioxidation, anti-apoptosis, and cell aggregation effects, thereby promoting MSC chondrogenesis.

**Figure 8:**
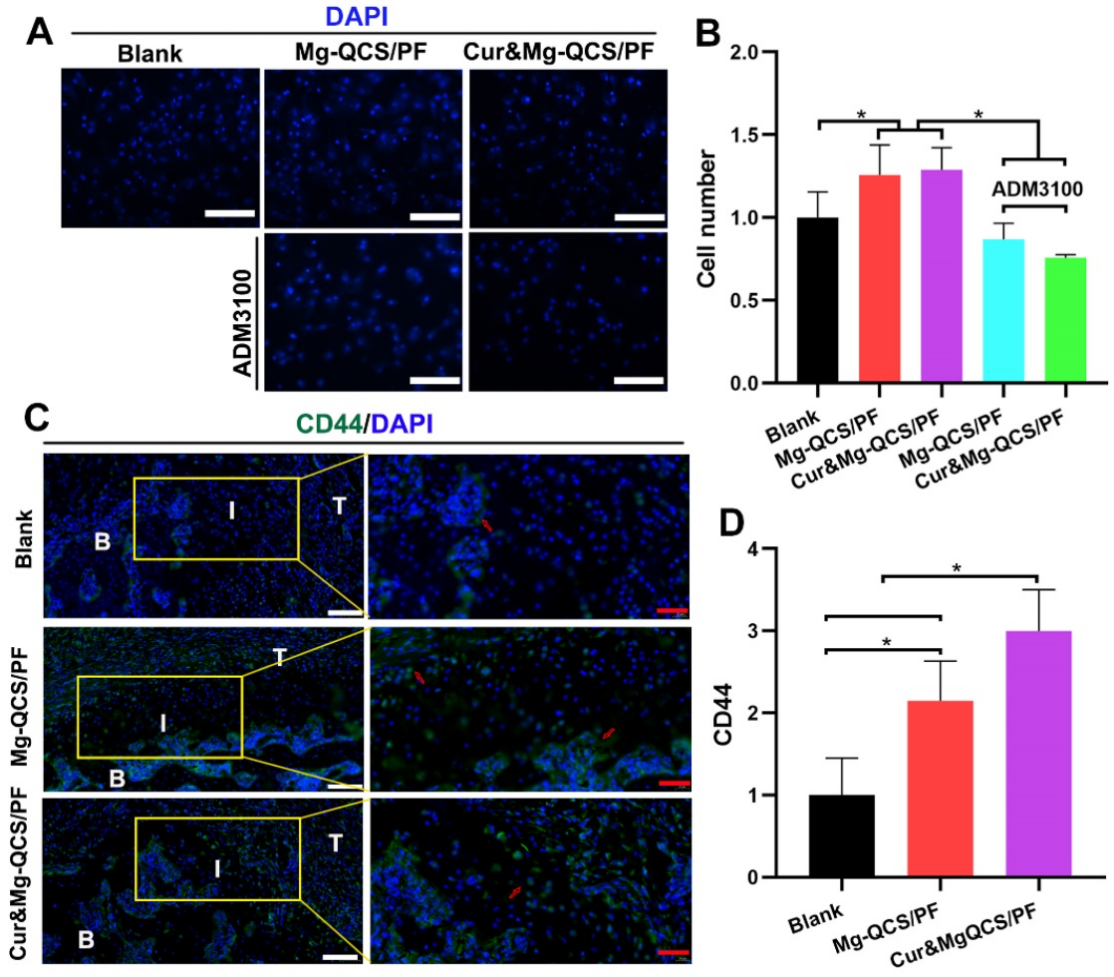
MSC recruitment by Mg^2+^-loaded hydrogels *in vitro* and *in vivo*. (A) Representative images of migrated BMSCs stained with DAPI. Scale bars: 100 µm. (B) Number of migrated cells (n = 3). (C) Representative images of repaired tendon stained for CD44. The red arrows indicate CD44-positive cells. White scale bars: 100 µm, red scale bars: 40 µm. (D) Number of CD44-positive cells (n = 6). The data was normalized to the blank group. *P < 0.05.

It is imperative to recruit MSCs to the defect site for cartilage regeneration, especially for rotator cuff tendon-to-bone healing 6, 59. Traditional chemotactic factors like SDF-1 have exhibited problems that make their clinical application difficult, such as poor efficiency, side effects at high dosages, and high costs 60. As shown in Figure 8A-B, Mg^2+^-doped hydrogels showed more obvious chemotaxis effects on BMSCs than no treatment. Additionally, the chemotaxis effect of Mg^2+^ was significantly diminished by ADM3100, suggesting that the mechanism of Mg^2+^ promotion of cell chemotaxis might be related to CXCR4 receptor 20. To further study cell aggregation by Mg^2+^ *in vivo*, the hydrogels were injected into the rat rotator cuff tear and repair model and MSCs were detected at 2 weeks after surgery. The cell surface marker CD44 was used to identify MSCs according to the previous literature 61. As shown in Figure 8C-D, significantly more CD44-positive cells were found around the repair site in the Mg^2+^-doped hydrogel groups compared to the blank group 2 weeks after implantation. These results confirmed the positive effects of Mg^2+^ released from the hydrogels on MSC recruitment *in vivo*.

### Cur&Mg-QCS/PF promoted rotator cuff healing via synergistic effects

**Figure 9:**
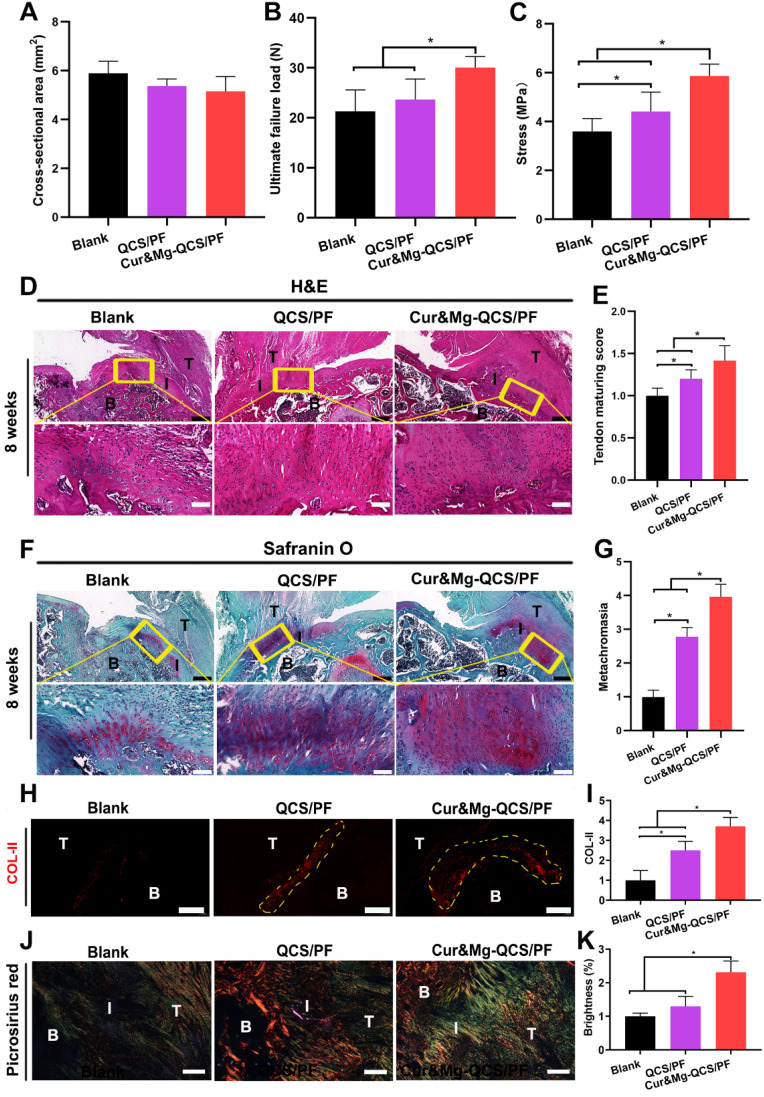
*In situ* regeneration of a native-like fibrocartilaginous interface between rotator cuff tendon and bone by Cur&Mg-QCS/PF. (A) Cross-sectional area, (B) ultimate failure load, and (C) stress of repaired tendon enthesis (n = 6). (D) Representative images of repaired tendon enthesis stained for H&E. Black scale bars: 400 µm, white scale bars: 100 µm. B: bone, I: interface, T: tendon. (E) Histological examination of repaired tendon (n = 6). (F) Representative images of repaired tendon enthesis stained with Safranin O. Black scale bars: 400 µm, white scale bars: 100 µm. B: bone, I: interface, T: tendon. (G) Metachromasia area stained by Safranin O (n = 6). (H) Representative images of repaired tendon enthesis stained for COL-II. The yellow circles indicate COL-II-positive areas. Scale bars: 200 µm. (I) Semi-quantitative analysis of COL-II immunofluorescence staining (n = 6). (J) Representative images of repaired tendon enthesis stained with picrosirius red. Scale bars: 200 µm. B: bone, I: interface, T: tendon. (K) Brightness of sections stained with picrosirius red (n = 6). Semi-quantitative data were normalized to the blank group. *P < 0.05.

As shown in Figure S9, dense and smooth native-like tissues were detected between tendon and bone in model rats treated with Cur&Mg-QCS/PF, while more scar-like tissues were observed in the blank group. In addition, the biomechanical properties of the regenerated rotator cuff were investigated using a materials test system 22. As shown in Figure S10, for all groups, failure occurred at the supraspinatus tendon-to-bone junction site. In addition, as shown in Figure 9A-C, the Cur&Mg-QCS/PF group exhibited significantly higher load of failure and stress values compared to the blank and QCS/PF groups. It is well known that the native transitional interface structure is essential to minimize stress concentration and to achieve effective load transfer from tendon to bone 62, 63. It is generally believed that rotator cuff injuries always lead to structural and functional deterioration 64, 65. Clinically, failure of surgically repaired torn rotator cuff tendons is probably due to the lack of sufficient resistance stress of the newly formed fibrovascular scar tissues within the gap between tendon and bone 66, 67. Hence, successful repair of rotator cuff ruptures relies on functional regeneration of the tendon-to-bone interface. The excellent biomechanical performance of the Cur&Mg-QCS/PF group indicated a strong tendon-to-bone connection associated with functional regeneration of a multi-tissue graded structure at the tendon-to-bone interface.

The natural tendon-to-bone attachment presents a fibrocartilaginous interface with a gradient from tendon to unmineralized fibrocartilage, mineralized fibrocartilage, and bone 68. Histological sections of repaired rotator cuff tendon tissues with H&E staining showed denser and more organized collagen fibers in the Cur&Mg-QCS/PF group at 8 weeks after surgery (Figure 9D) with higher tendon maturing scores (Figure 9E) than the blank and QCS/PF groups. Additionally, the extent of tendon repair was improved by Cur&Mg-QCS/PF, which may be related to the anti-inflammatory and matrix degradation reduction effects of curcumin. Newly formed fibrocartilaginous matrix rich in proteoglycan was also clearly observed at the tendon-to-bone junction in the Cur&Mg-QCS/PF group, which was evidenced by Safranin O staining, in contrast to the disrupted matrix with fewer proteoglycans in the blank and QCS/PF groups (Figure 9F-G). Consistent with this result, the Cur&Mg-QCS/PF group exhibited expression of collagen type II (COL-II), indicating a native-like COL-II-positive fibrocartilaginous gradient matrix, whereas newly formed fibrovascular scar tissues at the tendon insertion site showed a lack of COL-II expression in the blank and QCS/PF groups (Figure 9H-I). COL-II is a specific collagen molecule that distinguishes fibrocartilage from scar tissue; hence, it is considered a marker of fibrocartilage regeneration 6. Moreover, at 8 weeks post-surgery, the Cur&Mg-QCS/PF group showed densely aligned tendon collagen fibers at the tendon-to-bone insertion site, whereas a disorganized collagen structure was observed in the blank group (Figure 9J). In addition, significantly higher gray values of picrosirius red staining were seen at the tendon-to-bone interface in the Cur&Mg-QCS/PF group compared to the blank and QCS/PF groups (Figure 9K). These results indicated the capability of the composite hydrogel to improve fibrocartilage layer regeneration and collagen organization during the rotator cuff healing process, which are closely related to the functional reconstruction of a multi-tissue gradient interface.

Although various tissue engineering strategies have been developed to promote tendon-to-bone healing, it remains challenging to promote the formation of gradient interfaces, especially integrated fibrocartilage layers 69-71. Additionally, the previous literature only focused on the construction of bony insertion structures and lacked attention to repair of supraspinatus tendinous tissue. Loose and scarred supraspinatus tendinous tissue is not good for tendon-to-bone integration, which might explain the poor biomechanics of the tendon-to-bone interface after traditional tissue engineering repair. Therefore, it is still imperative to explore new tissue engineering strategies to improve rotator cuff healing. In recent years, the inflammatory status of rotator cuff injury has attracted increasing attention 10. We believe that the continuous inflammatory environment at the interface after injury impedes regeneration of the fibrocartilaginous interface and severely weakens the biological effects of traditional tissue engineering strategies. In this study, we constructed a protective microenvironment and a differentiation-inducing microenvironment by *in situ* release of curcumin and Mg^2+^ from hydrogels. This synergistic effect not only improved the formation of fibrocartilage matrix at the interface, but also promoted the repair of rotator cuff tendinous tissue, which was conducive to the integration of tendon and bone. Taken together, these results indicated that the engineered Cur&Mg-QCS/PF composite hydrogel has great potential to promote rotator cuff healing.

## Conclusion

A novel multifunctional hydrogel encapsulating curcumin and Mg^2+^ was developed and demonstrated to significantly promote rotator cuff healing. The composite hydrogel was injectable, adhesive, self-healing, and porous and exhibited suitable rheological properties. Cur&Mg-QCS/PF also showed sustained release of Mg^2+^ and curcumin. The composite hydrogel mediated *in situ* release of curcumin, which created a protective environment for stem cells and tendon tissue by inhibiting ROS and inflammatory cytokines. Additionally, Mg^2+^ released from the hydrogel promoted stem cell adhesion, migration, aggregation, and chondrogenic differentiation. Animal experiments further showed that the synergetic effects of curcumin and Mg^2+^ promoted regeneration of neo-fibrocartilage tissues and organized collagen fibers, resulting in strong biomechanical performance. Taken together, these results indicated that the engineered Cur&Mg-QCS/PF composite hydrogel has great potential to promote rotator cuff healing and shows potential value for clinical application.

## Supplementary Material

Supplementary figures and tables.
