# Supplementary material for: Synergistic enhancement of tendon-to-bone healing via anti-inflammatory and pro-differentiation effects caused by sustained release of Mg2+/curcumin from injectable self-healing hydrogels
Source: Theranostics. 2021 Apr 3;11(12):5911–25. doi: 10.7150/thno.56266 (PMC8058719; doi:10.7150/thno.56266)
Supplement: Supplementary file 1 — Supplementary figures and tables. [file thnov11p5911s1.pdf]

Supporting information

Synergistic enhancement of tendon-to-bone healing via anti-inflammatory and pro-differentiation effects caused by sustained release of Mg^{2+}/curcumin from injectable self-healing hydrogels

Baojun Chen [a, 1], Yongping Liang [b, 1], Jing Zhang [c, 1], Lang Bai [a], Meiguang Xu [a], Qian Han [a], Xuezhe Han [a], Jintao Xiu [a], Meng Li [a], Xiaoling Zhou [a], Baolin Guo [b, *], Zhanhai Yin [a, *]

a. Department of Orthopaedics, The First Affiliated Hospital of Xi'an Jiaotong University, Xi'an, 710061, China.

b. Frontier Institute of Science and Technology, and State Key Laboratory for Mechanical Behavior of Materials, and Key Laboratory of Shaanxi Province for Craniofacial Precision Medicine Research, College of Stomatology, Xi'an Jiaotong University, Xi'an, 710049, China.

c. Key Laboratory of Resource Biology and Biotechnology in Western China, Ministry of Education. School of Medicine, Northwest University, 229 Taibai North Road, Xi’an 710069, China.

* Corresponding author.

E-mail address: baoling@mail.xjtu.edu.cn (B. Guo), zhanhai.yin@mail.xjtu.edu.cn (Z. Yin)

[1] These authors contributed equally to this work.

Materials and methods

FT-IR spectroscopy

The IR spectra of QCS, PF127-CHO, QCS/PF, Mg-QCS/PF, Cur-QCS/PF, and Cur&Mg-QCS/PF were collected on a Nicolet 6700 FT-IR spectrometer (Thermo Scientific) over 650–4000 cm^{-1} [1].

Electron microscopy

To evaluate the morphology of the hydrogels, lyophilized hydrogel samples were sprayed with a thin gold layer and then imaged on a field emission scanning electron microscope (QUTAN FEG 250, FEI) according to a published protocol [2].

Adhesive strength test

The adhesive ability of the composite hydrogel to the host tissue was measured using fresh porcine skin following our previous work [1]. Briefly, the skin tissue was cut into 10 mm × 30 mm rectangles and then immersed into DPBS before use. 100 μL of Cur&Mg-QCS/PF solution was applied onto the surface of the porcine skin and then another piece of skin was put onto the hydrogel solution layer. The adhesive area was 10 mm × 10 mm. After the hydrogel completely formed, the adhesion properties were measured by a lap shear test on an Instron Materials Test System (MTS Criterion 43) equipped with a 50 N load cell at a rate of 5 mm/min.

Self-healing tests

To assess self-healing macroscopically, hydrogel disks were cut into tiny pieces and blended. The mixture was put into a cylindrical mold and left to heal for 2 h and then photographed. To quantitatively assess self-healing, hydrogel disks were prepared with

a diameter of 20 mm and a height of 1 mm. An alternate strain sweep test was performed at a fixed angular frequency (1 rad·s^{-1}). Amplitude oscillatory strains were switched from small strain (γ = 1.0%, 60 s for each interval) to large strain (γ = 500%, 60 s for each interval) and 3 cycles were carried out.

Rheological measurements

A rheometer (DHR-2, TA Instruments) was employed to measure the rheological properties of the hydrogels. 500 μL of hydrogel solution was placed between 20 mm diameter parallel plates with a gap of 1000 μm at a constant frequency of 1 rad/s and 1% strain. Time sweep tests were performed at 37 ℃. The mechanical properties of the hydrogels were further studied following our previous work [1]. Hydrogel samples were prepared with a cylindrical shape (~9 mm high, 12 mm in diameter) for compression tests at 25 °C. Cyclic compression tests were performed with a speed of 6 mm/min at 60% strain and then recovered to 0% strain with a speed of 6 mm/min. This cycle was repeated 20 times to evaluate the compression and recovery performance. All these tests were performed more than 5 times.

In vitro and in vivo degradation tests

For the in vitro degradation test, equivalent volumes of bulk hydrogel were immersed in 30 mL of PBS (pH = 7.4) at 37 ℃ with shaking at 100 rpm. At predetermined timepoints, the hydrogel samples were removed and rinsed with deionized water to remove salts. They were then dried in an oven at 60 ℃ for 48 h and weighed. The remaining weight ratio (%) was calculated as $(W_t – W_0) / W_0 \times 100\%$, where W_0 and W_t were the dry weights of the hydrogel before and after degradation. For the in vivo

degradation test, the hydrogel was implanted under the skin of rats. At predetermined timepoints, the hydrogel samples were removed and weighed. The remaining weight ratio was calculated using the same method as the in vitro degradation test.

In vitro curcumin and Mg^{2+} release assays

For the curcumin release assay, a similar method as our prior work was performed [1]. 200 μL of Cur&Mg-QCS/PF was prepared in a 4 mL centrifuge tube and allowed to completely gel. 3 mL of PBS (pH = 7.4) was pipetted into the tube at 37 °C with shaking at 100 rpm. At predetermined timepoints, 1 mL of the release buffer was removed for analysis and 1 mL of fresh buffer was added to maintain a constant volume. Curcumin released from the hydrogels was detected by its absorption at 426 nm (Lambda 35, PerkinElmer). For the Mg^{2+} release assay, the similar method was used but Mg^{2+} was detected using a blood Mg^{2+} concentration kit [3].

Cytocompatibility assays

The cytotoxicity of the hydrogels was investigated using a CCK-8 kit according to the manufacturer's instructions. Based on our prior work [2], hydrogel disks (5 mm in diameter, 0.5 mm thick) were prepared. 100 μL of BMSCs suspension (2.5×10^4 cells/mL) were seeded on 96-well plates. After overnight incubation, the cells were co-cultured with the hydrogel disk in complete growth medium supplemented with Dulbecco's Modified Eagle's Medium (Hyclone) containing 10% fetal bovine serum (BI) and 1% penicillin and streptomycin (Hyclone) for 1, 3, or 5 days. At each timepoint, 110 μL of CCK-8 was added and incubated at 37 °C for 4 hours. The optical density at 490 nm was measured using a microplate reader. All the tests were conducted in

triplicate. In addition, LIVE/DEAD® Viability/Cytotoxicity Kit was used to evaluate the viability of cells co-cultured with hydrogels. After 5 days of co-culture, the hydrogel disks and medium were removed and 110 μL of pre-diluted LIVE/DEAD® reagent was added. After incubation for 45 min, images were obtained under an inverted fluorescence microscope (Nikon, Ti-S).

Generation of the rat acute rotator cuff tear and repair model and implantation of hydrogels

Based on methods described in the literature [4], we constructed an animal model of hydrogel repair for injured tendons. Sprague Dawley rats (230–250 g, male) were anesthetized with 10% chloral hydrate with xylazine hydrochloride intraperitoneal injection. After skin preparation, a longitudinal incision was made over the shoulder joint. The deltoid muscle was separated by blunt dissection and the acromion was retracted to fully expose the rotator cuff tendon. The supraspinatus tendon was sharply detached from its insertion site at the greater tuberosity using a No.11 surgical blade. A 6-0 non-absorbable silk suture was used to pull the tendon to the greater tuberosity and fix it on the humerus cortex. For hydrogel-treated rats, 50 μL of hydrogel was injected around the repaired tendon. For blank rats, no treatment was given. After the skin was closed, the animals were allowed to move normally in the cage with free access to water and food.

Histological and immunofluorescence analysis of repaired tendon protection by Cur-QCS/PF

Harvested tendon tissues were fixed in 4% paraformaldehyde, embedded in paraffin,

and then sectioned into 4 μm slices. H&E and Masson's trichrome staining were used to examine the tissue morphology and ECM organization according to a previous study [4]. In addition, the tissue sections were analyzed for protein expression using immunofluorescence staining following standard published protocols [4]. Primary antibodies for IL-1β, TNF-α, SOD-1, MMP-9, and MMP-13 were used. Nuclei were counterstained with DAPI, and images were obtained using a fluorescence microscope. Positively stained area was quantified using NIH ImageJ software.

In vitro cell adhesion assay

Hydrogel extracts were prepared according to our previous work [2]. Then, rat BMSCs were suspended in 400 μL of hydrogel extract at a density of 1×10^5 cells/mL and seeded on 24-well plates for 10 min. BMSCs cultured in complete growth medium without hydrogel extract were used as control. After incubation, the cells were stained with DAPI. Then, images were obtained on an inverted fluorescence microscope (Nikon, Ti-S) and NIH ImageJ software was used to count the cells.

In vitro chemotaxis assay

To investigate the chemotaxis effects of the hydrogels, a transwell system was used. BMSCs suspended in 200 μL of serum-free medium at a density of 2.5×10^6 cells/mL were seeded in the upper chamber and 400 μL of hydrogel extract was added to the lower chamber. Growth medium was added to the lower chamber in the control group instead of hydrogel extract. After incubation for 24 h, the upper surface of the filter was scraped free of cells and debris. The cells that had migrated through the filter were stained with DAPI. Then, images were obtained on an inverted fluorescence

microscope (Nikon, Ti-S) and NIH ImageJ software was used to count the cells. Furthermore, rat BMSCs were also pretreated with ADM3100 (CXCR4-specific antagonist) to investigate the potential mechanism of Mg^{2+} on migration. Following a reported method [5], the BMSCs were pretreated with ADM3100 (10 μg/mL) for 2 h at 37 ℃. Cell migration was then investigated using the method described above.

In vitro chondrogenic differentiation and gene expression assays

To investigate the gene expressions of fibrocartilage markers (ACAN, COL2A1, SOX-9, Decorin) and growth factor (BMP-2), RT-qPCR analysis was conducted. Rat BMSCs were seeded on 6-well plates and co-cultured with hydrogels disks (30 mm in diameter, 0.5 mm thick) in chondrogenic medium. After 7 and 14 days of culture, total RNA was isolated using a fast 200 kit according to the manufacturer's instruction and then reverse transcribed into cDNA. qPCR was conducted with TB Green kit and the target gene was normalized to the housekeeping gene (GAPDH). The primers are listed in Table S1.

Table S1. Primer sequences

Primer	F	R
COL2A1	CTGGTTTGGAGAGACCATGAA	TGGACAGTAGACGGAGGAAA
SOX-9	GGAGGAAGTCGGTGAAGAATG	GAAGATGGCGTTAGGAGAGATG
ACAN	GGTGTCACTTCCCAACTATCC	GCATCACTTCACACCGATAGA
BMP-2	ATCCACTCCACAAACGAGAAA	CCACATCACTGAAGTCCACATA
Decorin	GCTAGCCTGAAAGGACTGAATAA	GCTTGTTGTTGTCCAAGTGAAG
GAPDH	ACTCCCATTCTTCCACCTTTG	CCCTGTTGCTGTAGCCATATT

Biomechanical evaluation of in vivo healing

The supraspinatus tendon-promixal humerus complex was harvested 8 weeks after surgery and stored at –80 ℃ for biomechanical tests. The samples were thawed at room temperature on the day of testing, and all redundant tissues were carefully removed by sharp dissection. Pull-to-failure analysis was conducted using the MTS tension test system according to prior studies with some modifications [2, 6]. First, the cross-sectional area of the tendon insertion was measured using a digital caliper. Then, the humerus and the free end of the supraspinatus tendon were positioned in the clamp and secured with a proper angle between tendon and bone. Tensile tests were done at a rate of 2 mm/min. Finally, the ultimate failure load and failure modes were recorded. The maximum stress was obtained by dividing the ultimate failure load by the initial cross-sectional area.

Histological analysis of in vivo healing

Rats were euthanized 8 weeks after surgery, and the supraspinatus tendon-proximal humeral complex was collected. The samples were fixed in 4% paraformaldehyde, embedded in paraffin, and then sectioned into 4 μm slices for further analysis. H&E, Safranin O, and picrosirius red staining were used to examine the tissue morphology, newly formed fibrocartilage, and collagen fiber organization at the tendon-to-bone interface. Additionally, the tendon maturing scoring system was used to evaluate the repaired tendons and their insertion [7, 8]. In this system, 7 parameters were included: vascularity, cellularity, proportion of parallel cells, and proportion of cells resembling tenocytes, proportion of fibers of large diameter characteristic of mature tendon fibers,

parallel fibers, and tendon-to-bone insertion remodeling. The weight of each parameter was added to obtain the total score, and groups were compared. Furthermore, the area specifically stained with Safranin O and the gray value of the tendon-to-bone junction in picrosirius red-stained sections were calculated using NIH ImageJ software.

Immunofluorescence analysis of in vivo healing

Tissue sections were analyzed for protein expression of COL-II by immunofluorescence staining. Nuclei were counterstained with DAPI, and images were acquired using a fluorescence microscope. The positively stained area was quantified using NIH ImageJ software.

Results and discussion

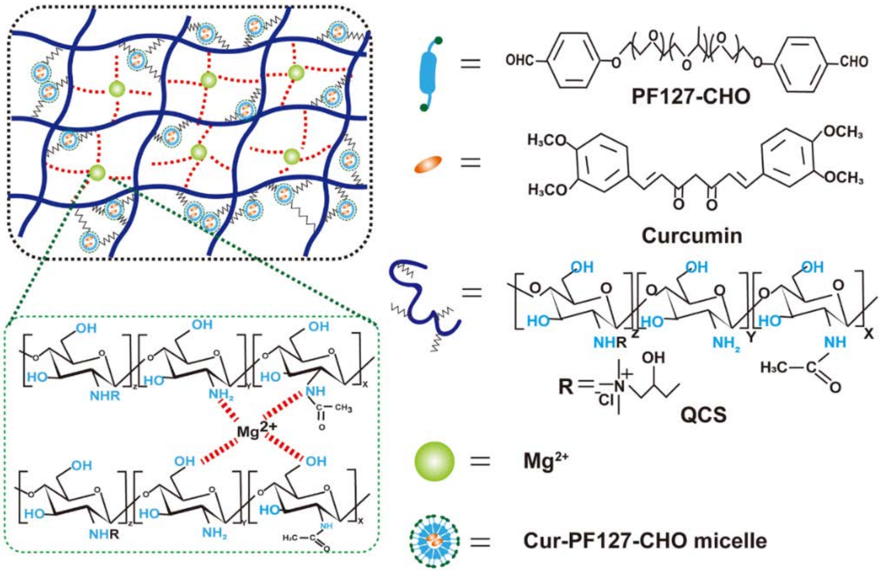

Figure S1. Schematic illustration and chemical structures of Cur&Mg-QCS/PF showing interactions between Mg^{2+} and –OH, $–NH_2$/NH groups in the QCS main chains.

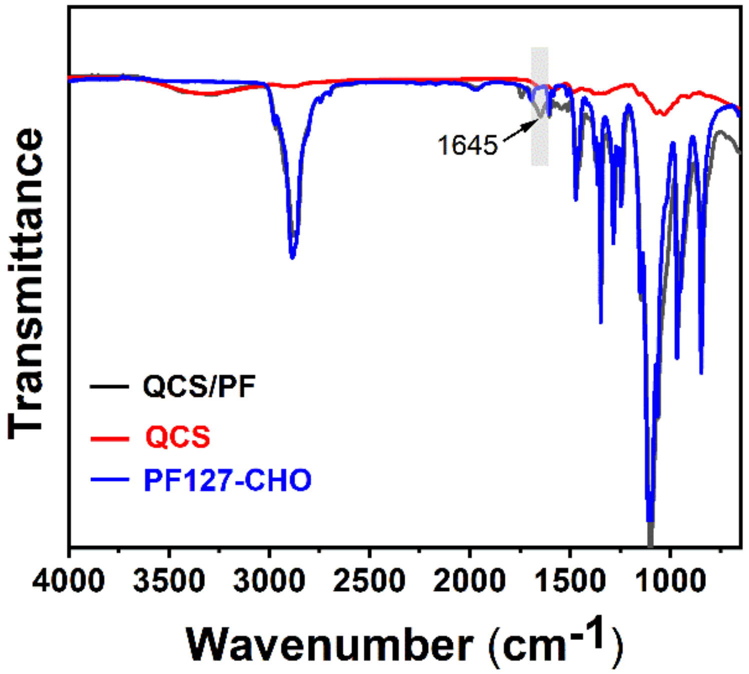

Figure S2. FT-IR spectra of QCS, PF127-CHO, and QCS/PF. A peak at 1645 cm^{-1} appeared in QCS/PF owing to the characteristic absorption of the newly formed Schiff base bond between amine groups of QCS and aldehyde groups of PF127-CHO.

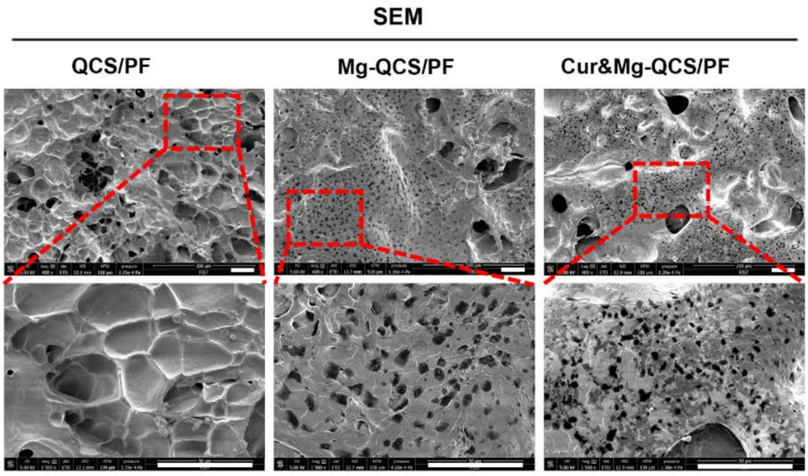

Figure S3. SEM images of the interior structures of the hydrogels. Scale bars: 50 μm.

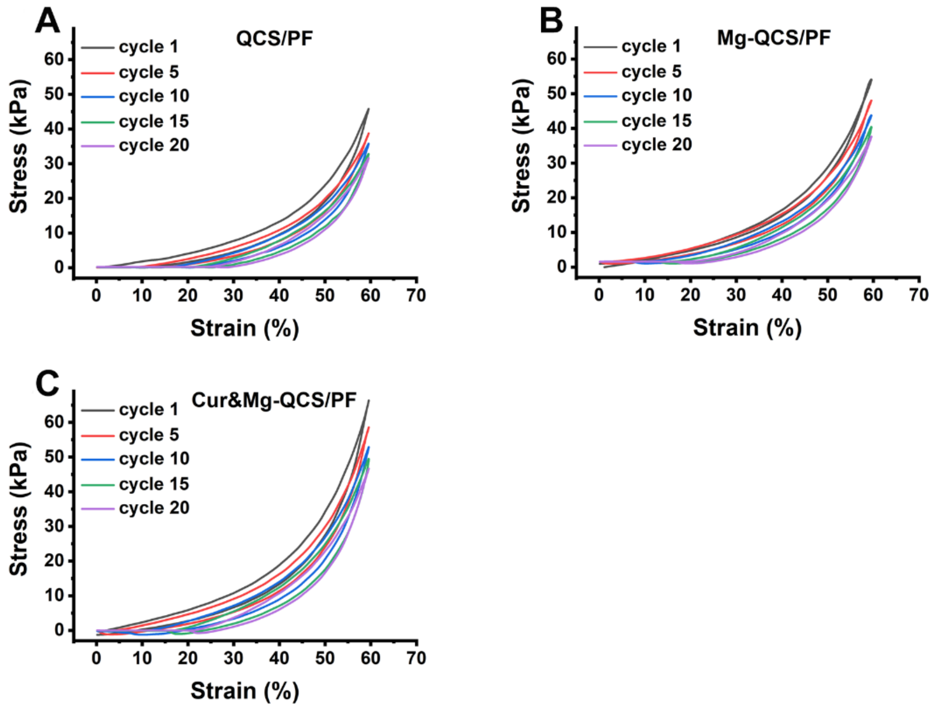

Figure S4. Cyclic compression tests of (A) QCS/PF, (B) Mg-QCS/PF, and (C) Cur&Mg-QCS/PF at a 60% strain with a speed of 6 mm/min. No breaking or strength decrease occurred in the hydrogels after 20 loading cycles, indicating their good robustness and resilience.

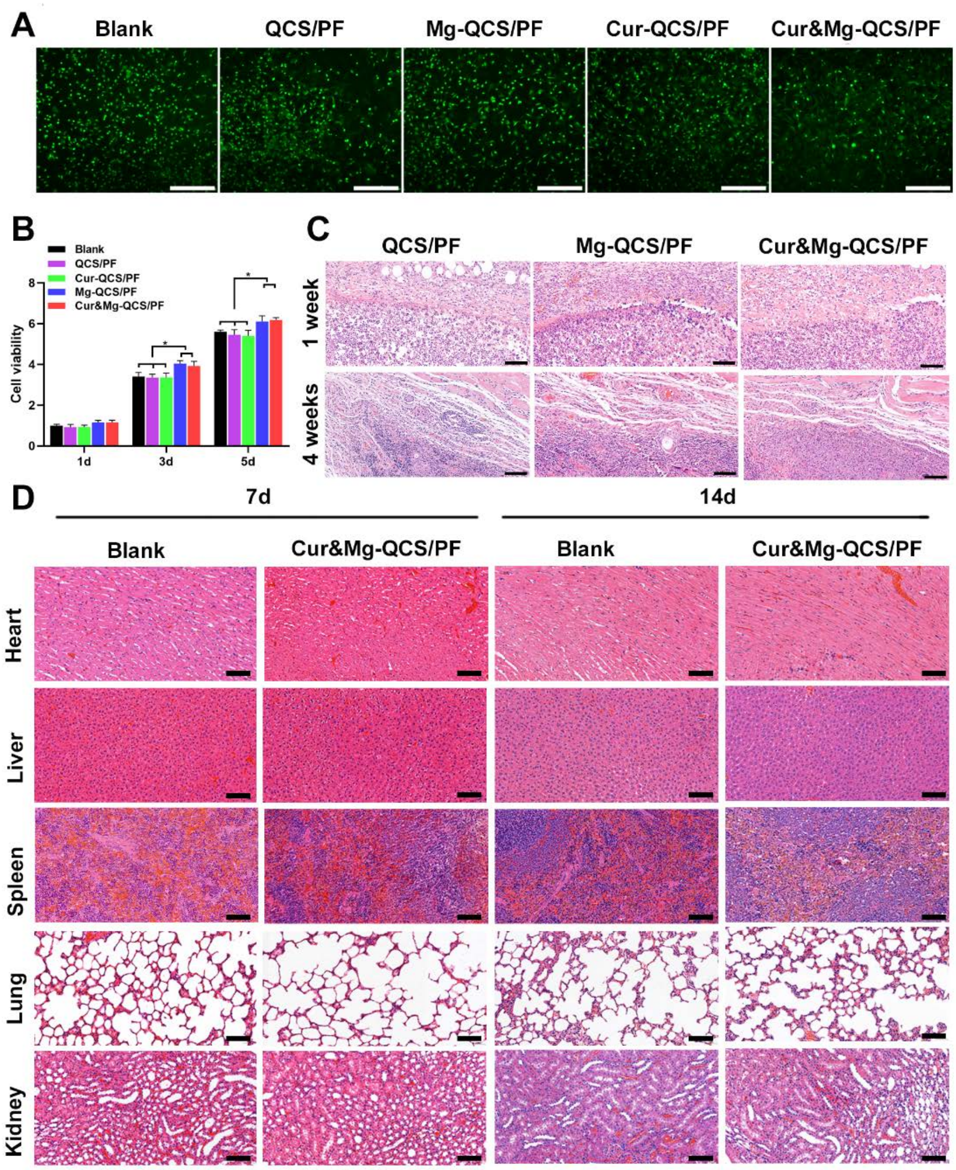

Figure S5. (A) LIVE/DEAD staining of rat BMSCs co-cultured with hydrogels for 5 days. Scale bars: 200 μm. (B) Cell viability of rat BMSCs directly contacting hydrogels (n = 3). The data were normalized to the blank group on day 1. *$P < 0.05$. (C) Images of subcutaneous tissues stained with H&E following subcutaneous implantation of the hydrogels. Scale bars: 100 μm. (D) Images of vital organs stained with H&E following subcutaneous implantation of the composite hydrogel. Scale bars: 100 μm.

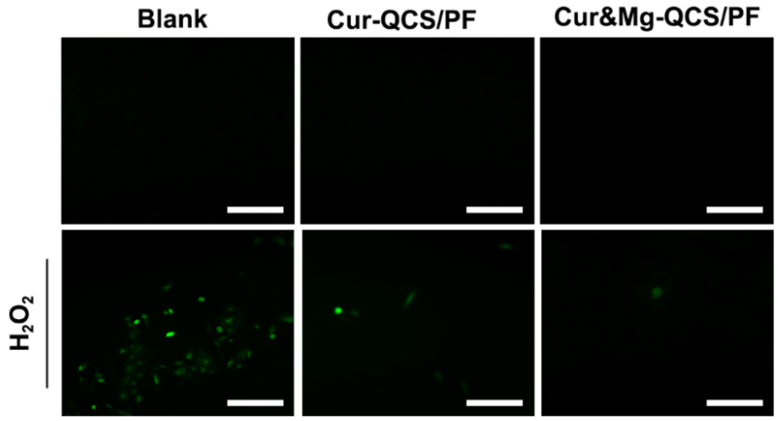

Figure S6. Representative images of ROS production induced by H_2O_2 and inhibited by curcumin in BMSCs detected by H2DCF-DA assay. Scale bar: 100 μm.

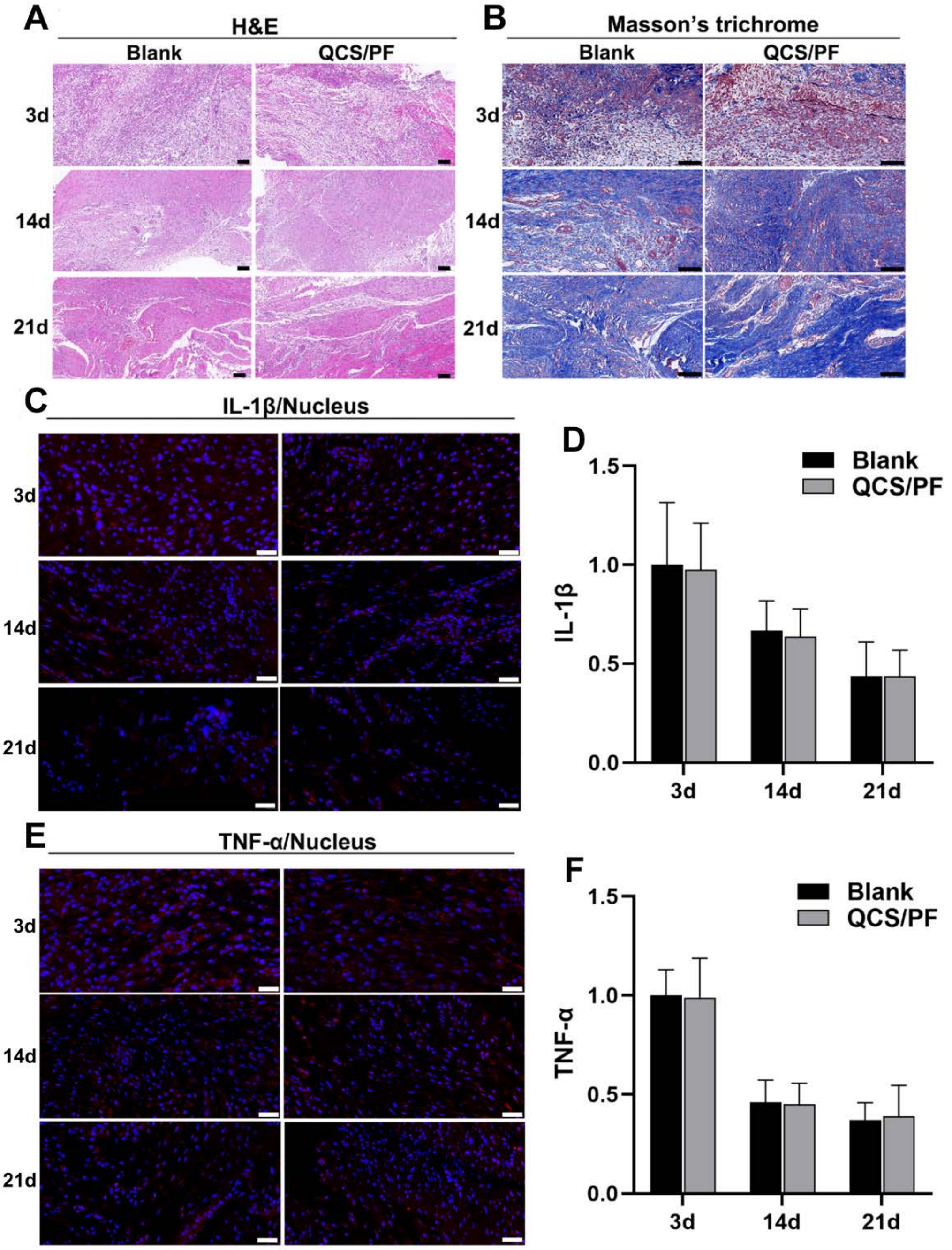

Figure S7. (A) Representative images of repaired tendon stained with H&E. Scale bars: 100 μm. (B) Representative images of repaired tendon stained with Masson's trichrome. Scale bars: 100 μm. (C) Representative images of repaired tendon stained for IL-1β. Scale bars: 40 μm. (D) Relative expression of IL-1β in tendon tissue (n = 6). (E) Representative images of repaired tendon stained for TNF-α. Scale bars: 40 μm. (F) Relative expression of TNF-α in tendon tissue (n = 6). The data was normalized to the

blank group on day 3. *$P < 0.05$.

In order to investigate whether QCS/PF hydrogel has anti-inflammatory effects, we further added a contrast experiment between the blank group and QCS/PF hydrogel group. Firstly, male Sprague Dawley rats (230–250 g) were used to construct a rotator cuff tear and repair model with the hydrogels injected into the tendon-to-bone interface. Secondly, rats were sacrificed 3, 14, and 21 days after surgery. The anti-inflammatory effects of QCS/PF hydrogel were investigated by histology and immunofluorescence assays (Figure S7-8).

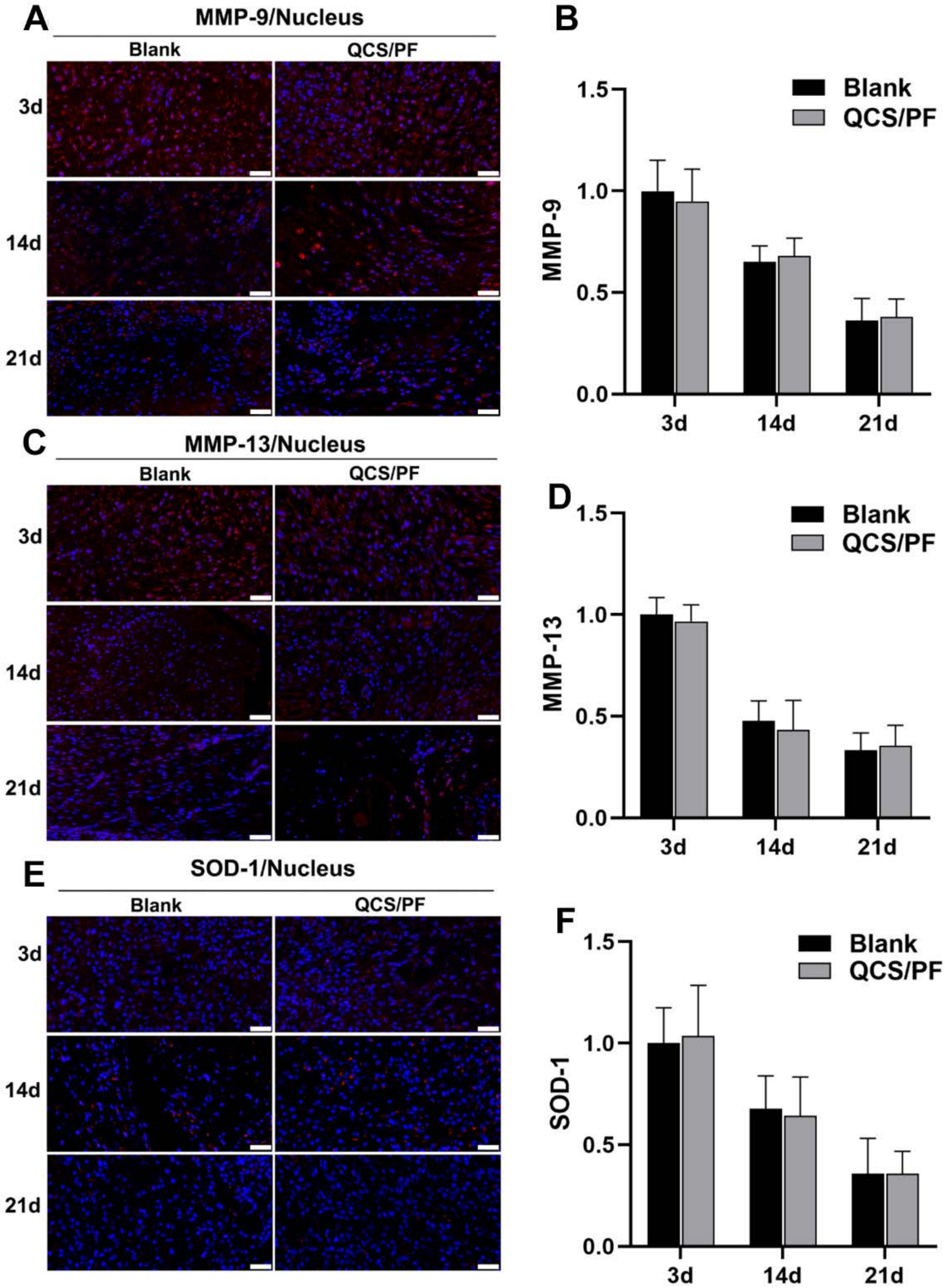

Figure S8. (A) Representative images of repaired tendon stained for MMP-9. Scale bars: 40 μm. (B) Relative expression of MMP-9 in tendon tissue (n = 6). (C) Representative images of repaired tendon stained for MMP-13. Scale bars: 40 μm. (D) Relative expression of MMP-13 in tendon tissue (n = 6). (E) Representative images of repaired tendon stained for SOD-1. Scale bars: 40 μm. (F) Relative expression of SOD-

1 in tendon tissue (n = 6). The data was normalized to the blank group on day 3. *P < 0.05.

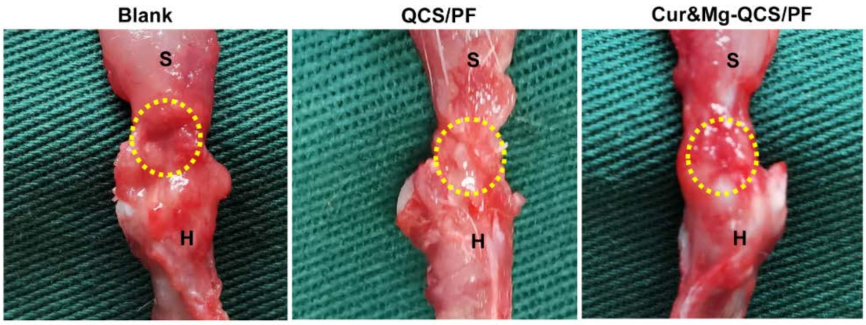

Figure S9. Gross observations of repaired tendon 8 weeks after surgery. S: supraspinatus, H: humerus.

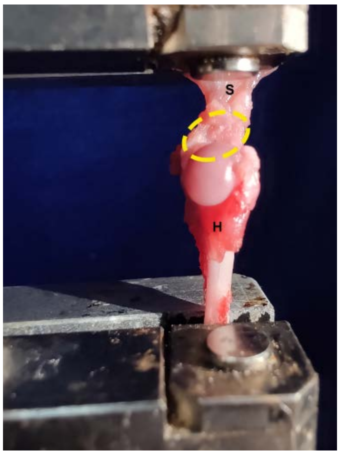

Figure S10. Failure occurred at the supraspinatus tendon-to-bone junction site (yellow circle). S: supraspinatus, H: humerus.

Reference

1. Qu J, Zhao X, Liang Y, Zhang T, Ma P, Guo B. Antibacterial adhesive injectable hydrogels with rapid self-healing, extensibility and compressibility as wound dressing for joints skin wound healing. Biomaterials. 2018; 183: 185-99.

2. Chen B, Liang Y, Bai L, Xu M, Zhang J, Guo B, et al. Sustained release of magnesium ions mediated by injectable self-healing adhesive hydrogel promotes fibrocartilaginous interface regeneration in the rabbit rotator cuff tear model. Chem Eng J. 2020; 396: 125335.

3. Zhang K, Lin S, Feng Q, Dong C, Yang Y, Li G, et al. Nanocomposite hydrogels stabilized by self-assembled multivalent bisphosphonate-magnesium nanoparticles mediate sustained release of magnesium ion and promote in-situ bone regeneration. Acta Biomater. 2017; 64: 389-400.

4. Thankam FG, Roesch ZK, Dilisio MF, Radwan MM, Kovilam A, Gross RM, et al. Association of inflammatory responses and ECM disorganization with HMGB1 upregulation and NLRP3 inflammasome activation in the injured rotator cuff tendon. Sci Rep. 2018; 8: 8918.

5. Zhang ZZ, Zhou YF, Li WP, Jiang C, Chen Z, Luo H, et al. Local administration of magnesium promotes meniscal healing through homing of endogenous stem cells: a proof-of-concept study. Am J Sports Med. 2019; 47: 954-67.

6. Zheng Z, Ran J, Chen W, Hu Y, Zhu T, Chen X, et al. Alignment of collagen fiber in knitted silk scaffold for functional massive rotator cuff repair. Acta Biomater. 2017; 51: 317-29.

7. Li X, Cheng R, Sun Z, Su W, Pan G, Zhao S, et al. Flexible bipolar nanofibrous membranes for improving gradient microstructure in tendon-to-bone healing. Acta Biomater. 2017; 61: 204-16.

8. Ide J, Kikukawa K, Hirose J, Iyama K, Sakamoto H, Mizuta H. Reconstruction of large rotator-cuff tears with acellular dermal matrix grafts in rats. J Shoulder Elbow Surg. 2009; 18(2): 288-95.
